# Association between adverse obstetric history and perinatal outcomes in singleton and twin pregnancies: a retrospective study

**DOI:** 10.3389/fmed.2026.1814198

**Published:** 2026-06-30

**Authors:** Wei-Zhen Tang, Xia Li, Hong-Yu Xu, Qin-Yu Cai, Ni-Ya Zhou, Yi-Fan Zhao, Hao-Wen Chen, Yue Tang, Fei Han, Tai-Hang Liu, Kai Ye

**Affiliations:** 1 Department of Obstetrics and Gynecology, Women and Children’s Hospital of Chongqing Medical University, Chongqing, China; 2 School of Basic Medical Sciences, Chongqing Medical University, Chongqing, China; 3 The Joint International Research Laboratory of Reproduction and Development, Chongqing Medical University, Chongqing, China; 4 Department of Obstetrics and Gynecology, Chongqing Health Center for Women and Children, Chongqing, China

**Keywords:** clinical characteristics, history of ectopic pregnancy, history of induced abortion, history of spontaneous abortion, twin pregnancy

## Abstract

**Objective:**

This study aimed to understand how adverse obstetric history affects perinatal outcomes in singleton and twin pregnancies, and to determine whether a twin pregnancy is a risk factor for adverse outcomes among women with an adverse obstetric history.

**Methods:**

This retrospective cohort study employed logistic regression to explore the relationship between adverse obstetric history, including spontaneous abortion (SAB), induced abortion (IA), and ectopic pregnancy (EP), and the occurrence of twin pregnancies. Multivariable analysis assessed the risk of adverse perinatal outcomes in both singleton and twin pregnancies among women with an adverse obstetric history. Clinical characteristics and outcome risks were compared between twin and singleton pregnancies with an adverse obstetric history. Additionally, the study investigated the association between the number of types of adverse obstetric history events and adverse perinatal outcomes in twin versus singleton pregnancies. Mediation causal analysis was then conducted to explore whether twin pregnancy mediated the relationship between adverse obstetric history and perinatal outcomes.

**Results:**

Among 17,180 pregnancies, 1,372 (7.99%) were twin pregnancies. After multivariable adjustment, the odds of twin pregnancies were 78% higher among women with a history of EP, while a history of IA was associated with a 49% lower odds of twin pregnancy. In singleton pregnancies, IA and EP were associated with a significantly increased risk of placenta accreta (OR: 1.29), placenta accreta with bleeding (OR: 1.62), fetal growth restriction (FGR) (OR: 1.87), and pelvic inflammation (OR: 1.69). In twin pregnancies, IA was associated with a significantly increased risk of gestational diabetes mellitus (GDM) (OR: 1.47), while EP was associated with a significantly increased risk of placenta previa (OR: 4.59) and placenta previa with bleeding (OR: 5.71). Compared with singleton pregnancies, twin pregnancies with an adverse obstetric history are associated with an increased risk of preeclampsia, FGR, intrahepatic cholestasis of pregnancy (ICP), preterm birth, and placenta previa with bleeding compared with singleton pregnancies. Specifically, the accumulation of three types of adverse obstetric history events is associated with a distinctly increased risk of GDM and placenta accreta with bleeding. Mediation analysis shows that twin pregnancy mediates the relationship between adverse obstetric history and adverse outcomes, with different mechanisms for IA and EP.

**Conclusion:**

Twin pregnancy is associated with an increased risk of adverse perinatal outcomes in women with a history of induced or ectopic pregnancy and appears to partly mediate the association between these histories and adverse outcomes.

## Introduction

Adverse obstetric histories, primarily comprising spontaneous abortion (SAB), induced abortion (IA), and ectopic pregnancy (EP), are globally prevalent (1), with approximately 30% of pregnancies ending in SAB (2, 3), over 43 million annual elective IAs (4), and EP occurring in ~2% of clinically recognized pregnancies, predominantly in the fallopian tubes (5–7). These histories are associated with multifactorial risks, including parental age, genetic predisposition, hormonal dysregulation, immune dysfunction, and environmental exposures (8, 9), while EP pathogenesis may involve impaired tubal transport and risk factors such as pelvic inflammatory disease, prior tubal EP, and smoking (10, 11). Advances in diagnostics, therapeutics, and assisted reproductive technology (ART) have improved subsequent intrauterine pregnancy rates in affected women (12); however, they face elevated risks of cardiovascular disease, venous thromboembolism (VTE) (13–15), metabolic disorders (e.g., type 2 diabetes mellitus) (16), and other long-term reproductive/health sequelae.

Emerging evidence highlights adverse obstetric histories as predictors of subsequent poor pregnancy outcomes. UK and Israeli studies associate SAB with recurrent PE, preterm birth, low-birth-weight infant, and GDM (17, 18), while IA correlates with preterm birth and low-birth-weight infant (19, 20), though some studies dispute its independent significance (21). Limited data suggest that a history of EP may elevate risks of preterm birth, low-birth-weight infant, placental abruption, and placenta previa in future pregnancies (22, 23). However, existing research predominantly focuses on singleton pregnancies with inconsistent conclusions.

Twin pregnancies, inherently high-risk due to uterine overdistension, placental dysfunction, and aberrant hemodynamics (24, 25), may exhibit heightened susceptibility to adverse perinatal outcomes when compounded with prior obstetric morbidity. Despite this, epidemiological and risk-stratification studies on twin pregnancies with adverse obstetric histories remain scarce. This retrospective cohort study aims to evaluate and compare the risks of adverse perinatal outcomes in singleton versus twin pregnancies with prior SAB, IA, or EP, and explore differences in the association between the number of adverse obstetric history types and perinatal outcomes between singleton and twin pregnancies. Findings will elucidate pregnancy-mode-specific risk profiles and inform tailored management strategies to mitigate perinatal complications in this vulnerable population.

## Materials and methods

### Ethical approval

This study was approved by the Women and Children’s Hospital of Chongqing Medical University (ID: 2024025). To safeguard patient privacy, all personally identifiable information was removed from the cases, and all acquired data were kept anonymous.

### Data collection

All data were extracted from the electronic medical records (EMR) system of the Women and Children’s Hospital of Chongqing Medical University by trained clinical personnel. The extracted data encompassed maternal demographics, obstetric histories, and pregnancy-related clinical parameters. Extracted variables included: (1) General characteristics (maternal age, pre-pregnancy BMI); (2) Obstetric history (prior adverse events: SAB, IA, EP); (3) Current pregnancy features (ART utilization); (4) Pregnancy complications [Gestational Diabetes Mellitus (GDM), Preeclampsia (PE), Fetal Growth Restriction (FGR), Intrahepatic Cholestasis of Pregnancy (ICP)]; and (5) Adverse perinatal outcomes (placenta accreta, placenta accreta with hemorrhage, placenta previa, placenta previa with hemorrhage, preterm delivery, cesarean section, and pelvic inflammatory disease). All of the above adverse outcomes were diagnosed or occurred during the pregnancy. Data abstraction followed standardized protocols with double-checking by independent reviewers to ensure accuracy and consistency.

### Study design

**Figure 1:**
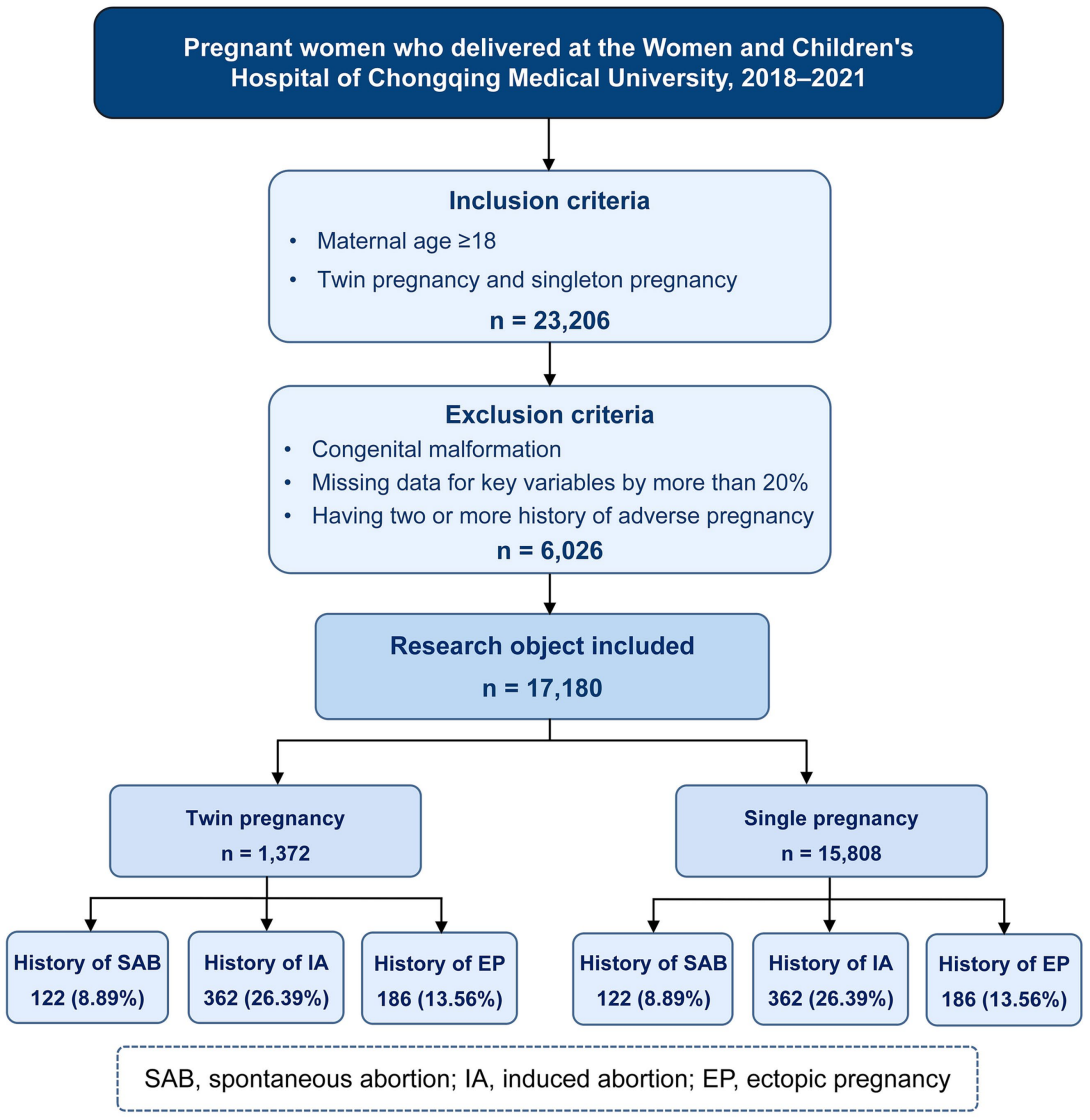
Flowchart of this retrospective cohort study.

This retrospective cohort study was conducted at the Affiliated Women and Children’s Hospital of Chongqing Medical University in China. The study retrospectively included pregnant women who regularly attended prenatal check-ups and delivered between 2017 and 2022. The inclusion criteria were as follows: (i) maternal age ≥18 years; (ii) singleton or twin pregnancies. The exclusion criteria were as follows: (i) congenital malformations; (ii) missing data for key variables by more than 20%. Each pregnant woman was uniquely identified by her name and medical record number. For those who had multiple deliveries during the study period, only the most recent delivery was included in the analysis to ensure the independence of observations. A total of 23,206 pregnant women met the inclusion criteria. After applying the exclusion criteria, 17,180 cases were finally included in this study (Figure 1). We used the STROBE (Strengthening the Reporting of Observational Studies in Epidemiology) guidelines for cohort studies.

### Exposure and outcome

Adverse obstetric history, as the primary explanatory variable, was categorized into SAB history, IA history, EP history, and no adverse obstetric history. These categories were not mutually exclusive, allowing each adverse history type to be considered independently while preserving the full clinical complexity of co-occurring events. This non-mutually exclusive classification helps avoid selection bias and loss of statistical power that would result from restricting analyses to single-history subgroups. In this study, induced abortion is defined as the deliberate termination of a pregnancy before 28 weeks of gestation or when the fetus weighs less than 1,000 g and cannot live independently outside of the womb (26).

The main outcomes of this study were adverse perinatal outcomes, including placental abnormalities, GDM, PE, FGR, ICP, preterm delivery, cesarean section, and pelvic inflammation. Placental abnormalities included placenta previa, placenta previa with bleeding, placenta accreta, and placenta accreta with bleeding. Placenta previa refers to the condition where the lower edge of the placenta reaches or covers the internal os of the uterus, with the internal os being below the fetal presenting part. The diagnosis of placenta previa was confirmed in the third trimester via transvaginal ultrasound, the gold standard, and was defined as the placental edge covering or lying within 2 cm of the internal cervical os. Placenta accreta is defined as the penetration of placental villi into the myometrial layer of the uterine wall. Pregnancies with risk factors for placenta accreta—including a diagnosis of placenta previa, a history of cesarean section, or other uterine surgery—underwent stringent screening based on ultrasonographic findings, in line with established guidelines (27–29). Magnetic resonance imaging (MRI) was employed as an adjunct in complex cases, such as suspected invasion of adjacent pelvic organs or when ultrasound assessment was indeterminate. The final diagnosis of placenta accreta was established either by (i) histopathological confirmation, the definitive standard based on microscopic identification of abnormal villous adherence (30), or (ii) clinical and intraoperative findings in accordance with the International Federation of Gynecology and Obstetrics (FIGO) guidelines when hysterectomy was not performed (31).

### Statistical analysis

To describe the sociodemographic characteristics of the study population, continuous variables were compared using Student’s independent t-test or Mann–Whitney U test based on the assumption of normality, with results reported as mean ± standard deviation (SD) or median (interquartile range, IQR). For categorical variables, results were presented as numbers and percentages, and comparisons were made using the chi-square test or Fisher’s exact test.

To examine the likelihood of subsequent twin pregnancies in women with adverse obstetric history, univariate and multivariate logistic regression analyses were conducted, adjusting for factors including maternal age, Pre-pregnancy Body Mass Index (PBMI), ART, nulliparity, age at menarche (the key indicator influencing women’s long-term reproductive and metabolic health), menstrual cycle (means the time from the first day of a menstrual period to the day before the next menstrual period), menstrual duration, endocrine system diseases (Diabetes mellitus, thyroid disorders, and pituitary prolactinoma requiring pharmacotherapy), history of smoking, and history of alcoholism (may influence ovulation and perinatal outcomes through mechanisms such as interference with the endocrine axis). Furthermore, we separately assessed the risk of adverse perinatal outcomes associated with adverse obstetric history in twin and singleton pregnancies and explored the impact of twin pregnancy on perinatal outcomes among those with adverse obstetric history. Then we grouped pregnant women based on the number of adverse obstetric history types (1, 2, or 3 types) to assess the cumulative effect. Subsequently, the relationship between this variable and adverse perinatal outcomes was analyzed separately in the single and twin pregnancy cohorts. Additionally, the impact of twin pregnancies on perinatal outcomes was further explored within subgroups defined by the number of types of adverse obstetric histories. Finally, causal mediation analysis was performed using the “mediation” package to estimate the effect of adverse obstetric history on perinatal outcomes mediated through twin pregnancy. All statistical analyses were conducted using SPSS software version 26.0 and R version 4.2.3 (The R Foundation for Statistical Computing, Vienna, Austria). A two-tailed *p*-value of <0.05 was set as the level of statistical significance for all tests, and the specific principles of mediation analysis are shown in Supplementary Figure S1.

## Results

### Baseline characteristics of the cohort population

In this study, after applying the exclusion criteria, a total of 17,174 pregnancies were included in the final analysis (see Figure 1). Among these, 15,808 (92.01%) were singleton pregnancies and 1,372 (7.99%) were twin pregnancies. The analysis of baseline characteristics revealed that the proportion of women undergoing ART (*p* < 0.001), nulliparity (p < 0.001), and a history of alcoholism (*p* = 0.026) was significantly higher in the twin pregnancy group, which may be potential risk factors for twin pregnancies. In terms of age, women with twin pregnancies were generally older and had a higher PBMI (Age: 30.98, IQR 28.61–33.49 vs. 30.58, IQR 27.60–33.92, *p* = 0.006; PBMI: 21.51, IQR 20.06–23.53 vs. 21.23, IQR 19.10–23.73, *p* < 0.001) (Table 1). Additionally, there were significant differences in the proportion of endocrine system diseases and menstrual cycle (*p* < 0.001). However, no statistical differences were found in the age at menarche, menstrual duration, and proportion of history of smoking between the two groups. Notably, the proportion of EP in adverse obstetric histories was significantly higher in the twin pregnancy group (13.56%) compared with the singleton pregnancy group (6.17%, *p* < 0.001). Meanwhile, the proportion of IA was lower in the twin pregnancy group (26.39%) compared with the singleton pregnancy group (42.97%, *p* < 0.001). The baseline characteristics of those with and without an adverse obstetric history are detailed in Supplementary Table S1.

**Table 1:** Demographic and clinical characteristics of women with singleton and twin pregnancies.

Characteristic [*N* (%)]	Twin pregnancy (*n* = 1,372)	Singleton pregnancy (*n* = 15,808)	Statistic (χ2)	*p* value
Age (Years)	30.98 [28.61, 33.49]	30.58 [27.60, 33.92]	−2.953	0.003*
PBMI, M (Q₁, Q₃)	21.51 [20.06, 23.53]	21.23 [19.10, 23.73]	−4.804	<0.001*
ART, *n* (%)	298 (21.72)	303 (1.92)	1466.559	<0.001*
Nulliparity, *n* (%)	1,185 (86.37)	10,081 (63.77)	285.609	<0.001*
Age at menarche	12.00 [11.00, 13.00]	12.00 [11.00, 13.00]	−1.385	0.146
Menstrual cycle	28.00 [28.00, 28.00]	28.00 [28.00, 29.00]	2.032	0.024*
Menstrual duration	5.00 [4.00, 6.00]	5.00 [4.00, 6.00]	−1.640	0.091
Endocrine system diseases	306 (22.30)	4,559 (28.84)	26.573	<0.001*
History of smoking, *n* (%)	26 (1.90)	435 (2.77)	3.609	0.057
History of alcoholism, *n* (%)	186 (13.70)	2,493 (15.99)	4.954	0.026*
History of cesarean section, *n* (%)	3 (0.219)	593 (3.751)	47.045	<0.001*
History of adverse pregnancy, *n* (%)				
SAB	122 (8.89)	1,404 (8.88)	0.000	0.989
IA	362 (26.39)	6,792 (42.97)	142.819	<0.001*
EP	186 (13.56)	975 (6.17)	109.388	<0.001*

PBMI, pre-pregnancy body mass index; ART, assisted reproductive technology; SAB, spontaneous abortion history; IA, induced abortion history; EP, ectopic pregnancy history.

^*^p < 0.05.

### Association between adverse obstetric history and the risk of twin pregnancy

To investigate the relationship between adverse obstetric history and the risk of twin pregnancy, we initially conducted an unadjusted logistic regression analysis. The results indicated that women with a history of EP had a significantly increased likelihood of subsequent twin pregnancies, with an OR of 2.39 (95% CI 2.02–2.82). Conversely, women with a history of IA had a significantly lower likelihood of twin pregnancies, with an OR of 1.78 (95% CI 1.47–2.15) (Table 2).

**Table 2:** The association between adverse obstetric history and twin pregnancy.

Adverse obstetric history	Univariate analysis [OR(95%CI)]	*p* value	Multivariate analysis [aOR^a^(95%CI)]	*p* value
SAB	1.00 (0.83, 1.22)	0.989	1.14 (0.91,1.42)	0.254
IA	0.48 (0.42, 0.54)	<0.001*	0.65 (0.55,0.77)	<0.001*
EP	2.39 (2.02, 2.82)	<0.001*	2.17 (1.77,2.66)	<0.001*

SAB, spontaneous abortion history; IA, induced abortion history; EP, ectopic pregnancy history.

^a^Adjusting for factors including maternal age, PBMI, ART, nulliparity, age at menarche, menstrual cycle, menstrual duration, endocrine system diseases, history of smoking, history of alcoholism, and history of cesarean section.

*p < 0.05.

After adjusting for potential confounding factors, the statistical results showed that the association between EP and twin pregnancy remained significant, with the OR slightly decreasing to 2.17 (95% CI 1.77–2.66). This continued to highlight the significant association between EP and twin pregnancy. On the other hand, the OR for IA increased slightly to 0.65 (95% CI 0.55–0.77), further emphasizing its significant association with a reduced risk of twin pregnancy. These findings underscore the clinically significant impact of adverse obstetric history, specifically EP and IA, on the subsequent risk of twin pregnancies (Table 2).

### Clinical characteristics and perinatal outcomes of adverse obstetric history in singleton and twin pregnancies

Further statistical analysis of the clinical characteristics of singleton and twin pregnancies with and without adverse obstetric history revealed the following findings: Compared with twin pregnancies, singleton pregnancies with a history of SAB were typically associated with higher age, higher BMI, and differences in menstrual duration. Additionally, a history of alcoholism was more prevalent among women with twin pregnancies and SAB (Supplementary Table S2). Regarding IA, singleton pregnancies were more likely to have higher BMI, use of ART, different age at menarche, and endocrine system diseases. For both singleton and twin pregnancies, women with IA were generally older, had a lower proportion of primigravida, and exhibited differences in menstrual cycle, menstrual duration, and history of smoking (Supplementary Table S3). Singleton pregnancies with a history of EP typically showed higher BMI, a higher proportion of endocrine system diseases, and differences in menstrual cycle and duration. In contrast, twin pregnancies were more likely to have a history of alcoholism and a lower proportion of primigravida. Regardless of singleton or twin pregnancies, women with EP were generally older and had a higher proportion of ART use (Supplementary Table S3).

**Figure 2:**
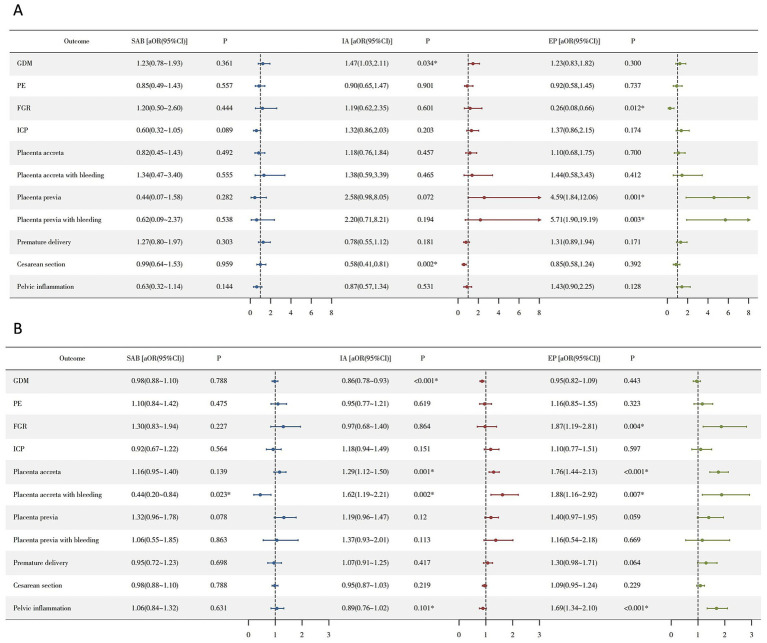
The impact of adverse obstetric history on the risk of adverse perinatal outcomes in twin and singleton pregnancies. **(A)** Twin pregnancy; **(B)** singleton pregnancy. The adjusted OR value for SAB [aOR(95%CI)] is calculated with Non-SAB as the reference. The adjusted OR value for IA [aOR(95%CI)] is calculated with Non-IA as the reference. The adjusted OR value for EP [aOR(95%CI)] is calculated with Non-EP as the reference. Adjusting for factors including maternal age, PBMI, ART, nulliparity, age at menarche, menstrual cycle, menstrual duration, endocrine system diseases, history of smoking, and history of alcoholism. *p < 0.05.

The risk of adverse perinatal outcomes associated with an adverse obstetric history was further analyzed separately for singleton and twin pregnancies. Specifically, IA and EP in singleton pregnancies significantly increased the risk of placenta accreta and placenta accreta with bleeding, with ORs of 1.29 (95% CI: 1.12–1.50) and 1.62 (95% CI: 1.19–2.21) for IA, and 1.76 (95% CI: 1.44–2.13) and 1.88 (95% CI: 1.16–2.92) for EP, respectively. Additionally, IA significantly increased the risk of FGR and pelvic inflammation, with ORs of 1.87 (95% CI: 1.19–2.81) and 1.69 (95% CI: 1.34–2.10), respectively (Figure 2; Supplementary Table S5). For twin pregnancies, IA only significantly increased the risk of GDM, with an OR of 1.47 (95% CI: 1.03–2.11), while EP significantly increased the risk of placenta previa and placenta previa with bleeding, with ORs of 4.59 (95% CI: 1.84–12.06) and 5.71 (95% CI: 1.90–19.19), respectively. The specific numbers and proportions of adverse perinatal outcomes associated with adverse obstetric history in singleton and twin pregnancies are detailed in Supplementary Table S6.

### Clinical characteristics and perinatal outcomes of singleton and twin pregnancies with an adverse obstetric history

A deep dive into the clinical characteristics of singleton and twin pregnancies with adverse obstetric history and their impact on adverse perinatal outcomes revealed that, among women with adverse obstetric history, twin pregnancies had significantly higher proportions of ART use and primigravida. Moreover, when comparing twin pregnancies with SAB to singleton pregnancies with SAB, menstrual duration was significantly different in twin pregnancies. For IA, age at menarche was also significantly different in twin pregnancies compared with singleton pregnancies. Additionally, a history of alcoholism was significantly higher in twin pregnancies with EP than in singleton pregnancies.

Further analysis showed that women with a history of pregnancy loss (including SAB and IA) in twin pregnancies had a higher risk of GDM, PE, FGR, ICP, placenta accreta with bleeding, and preterm delivery compared with singleton pregnancies. Specifically, in women with SAB, the incidence rates of PE, FGR, ICP, placenta accreta, placenta accreta with bleeding, preterm delivery, and cesarean section were significantly higher in twin pregnancies than in singleton pregnancies. After adjusting for potential confounders, multivariate logistic regression analysis further confirmed these findings. Compared with singleton pregnancies, the relative risks for PE, FGR, ICP, placenta accreta with bleeding, preterm delivery, and cesarean section in twin pregnancies with SAB were 3.05 (95% CI: 1.69–5.30), 3.08 (95% CI: 1.22–7.04), 4.40 (95% CI: 2.24–8.24), 2.97 (95% CI: 0.77–10.54), 8.45 (95% CI: 5.11–13.87), and 1.77 (95% CI: 1.19–2.66), respectively. Additionally, the risk of GDM was significantly higher (aOR: 1.11, 95% CI: 1.08–1.14), although the risk of placenta accreta with bleeding was no longer significant (Supplementary Table S7).

For twin pregnancies with IA, the incidence rates of the aforementioned adverse outcomes were also higher (PE 17.40% vs. 3.81%; FGR 6.63% vs. 1.22%; ICP 17.96% vs. 3.64%; placenta accreta 17.13% vs. 10.03%; placenta accreta with bleeding 3.87% vs. 1.50%; preterm delivery 25.97% vs. 4.76%; cesarean section 57.46% vs. 48.73%). Moreover, the risks of GDM, placenta previa with bleeding, and pelvic inflammation were significantly higher (GDM 43.92% vs. 35.41%; placenta previa with bleeding 2.76% vs. 0.97%; pelvic inflammation 16.30% vs. 5.88%). Further logistic regression analysis confirmed that the relative risks for GDM, PE, FGR, ICP, placenta accreta, placenta accreta with bleeding, placenta previa with bleeding, preterm delivery, and pelvic inflammation in twin pregnancies with IA were 1.47 (95% CI: 1.17–1.85), 5.35 (95% CI: 3.85–7.34), 5.87 (95% CI: 3.51–9.46), 6.35 (95% CI: 4.60–8.66), 1.64 (95% CI: 1.20–2.21), 2.27 (95% CI: 1.16–4.10), 3.80 (95% CI: 1.81–7.17), 6.71 (95% CI: 5.06–8.85), and 2.45 (95% CI: 1.75–3.38), respectively. For twin pregnancies with EP, the risks of PE, ICP, placenta previa with bleeding, preterm delivery, cesarean section, and pelvic inflammation were significantly higher. The relative risks for PE, ICP, placenta previa with bleeding, preterm delivery, cesarean section, and pelvic inflammation in twin pregnancies with EP were 3.01 (95% CI: 1.80–4.97), 6.88 (95% CI: 4.13–11.46), 8.47 (95% CI: 3.15–23.13), 6.66 (95% CI: 4.34–10.25), 1.44 (95% CI: 1.02–2.03), and 1.97 (95% CI: 1.26–3.01), respectively. These findings indicate that twin pregnancies with an adverse obstetric history face a significantly higher risk of adverse perinatal outcomes compared with singleton pregnancies. This finding holds significant importance for clinical management and risk assessment (Supplementary Table S7).

### Association between the number of adverse obstetric history types and adverse perinatal outcomes in twin versus singleton pregnancies

To analyze the combined impact of adverse obstetric histories, pregnant women were categorized based on the number of adverse obstetric events (1, 2, or 3 types). The relationship between the number of adverse obstetric history types and the risk of twin pregnancies was then examined. Adjusted logistic regression analysis revealed that women with three types of adverse obstetric histories (SAB, IA, and EP) had a significantly higher likelihood of subsequent twin pregnancies, with an OR of 2.45 (95% CI 1.25–4.58) (Supplementary Table S11).

**Figure 3:**
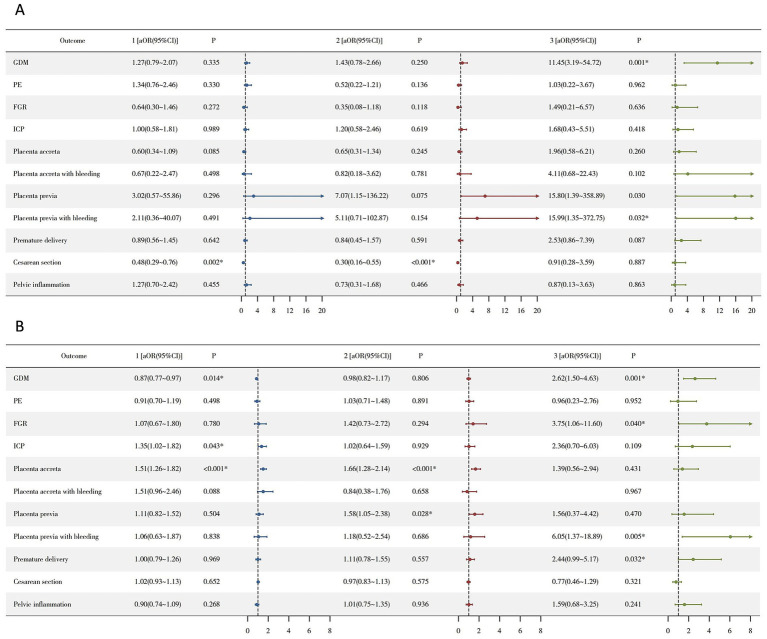
The impact of the number of types of adverse obstetric history on the risk of adverse perinatal outcomes in twin and singleton pregnancies. **(A)** Twin pregnancy; **(B)** singleton pregnancy. 1/2/3 [aOR (95% CI)] indicates the adjusted odds ratio for women with 1/2/3 types of adverse obstetric history compared with those without any adverse obstetric history. Models were adjusted for maternal age, PBMI, ART, nulliparity, age at menarche, menstrual cycle, menstrual duration, endocrine system diseases, history of smoking, and history of alcoholism. *p < 0.05.

The risk of adverse perinatal outcomes associated with the number of adverse obstetric history types was further analyzed separately for singleton and twin pregnancies. Specifically, in singleton pregnancies, three types of adverse obstetric histories significantly increased the risk of GDM, FGR, and placenta accreta with bleeding. The ORs were 2.62 (95% CI: 1.50–4.63) for GDM, 3.75 (95% CI: 1.06–11.60) for FGR, and 6.05 (95% CI: 1.37–18.89) for placenta accreta with bleeding, respectively. The presence of two types of adverse obstetric histories significantly increased the risk of placenta accreta (OR: 1.66, 95% CI: 1.28–2.14) and placenta previa (OR: 1.58, 95% CI: 1.05–2.38). For twin pregnancies, three types of adverse obstetric histories only significantly increased the risk of GDM (OR:11.45, 95% CI: 3.19–54.72) and placenta accreta with bleeding (OR: 15.99, 95% CI: 1.35–372.75). The presence of two types of adverse obstetric histories significantly decreased the risk of cesarean section (OR: 0.30, 95% CI: 0.16–0.55), while the association between the combination of all three adverse obstetric histories and cesarean section was not significant. The specific numbers and proportions of adverse perinatal outcomes associated with the number of adverse obstetric history types in singleton and twin pregnancies are detailed in Figure 3 and Supplementary Table S12.

Further analysis showed that women with three types of adverse obstetric histories (including SAB, IA, and EP) in twin pregnancies had a higher risk of ICP, preterm delivery, and cesarean section compared with singleton pregnancies. The ORs were 4.69 (95% CI: 1.00–22.32) for ICP, 7.37 (95% CI: 2.17–26.36) for preterm delivery, and 3.79 (95% CI: 1.20–14.60) for cesarean section, respectively. For women with two types of adverse obstetric histories, twin pregnancies had a higher risk of ICP, placenta accreta with bleeding, placenta previa with bleeding, and preterm delivery compared with singleton pregnancies (Supplementary Table S13).

### Analysis of twin pregnancy as a mediator between adverse obstetric history and pregnancy outcomes

Mediation analysis was conducted to explore the role of twin pregnancy as a mediator between adverse obstetric history and various pregnancy outcomes, including GDM, PE, ICP, placenta accreta, preterm delivery, and cesarean section. Specifically, for SAB, both the total effect and direct effect on GDM were significant (*p* = 0.006), while the indirect effect was not significant (*p* = 0.928), indicating that the impact of SAB on GDM does not pass through twin pregnancy as a mediating variable (Supplementary Table S14). Similarly, the mediation effects of SAB on other outcomes were also not significant (Supplementary Tables S15–S19).

In contrast, for IA, the total effect and direct effect on GDM were also significant (*p* < 0.001), but the indirect effect was not significant (*p* = 0.436) (Supplementary Table S20). For ICP and preterm delivery, the total effect and indirect effect of IA were significant (*p* < 0.05), but the direct effect was not significant (ICP, *p* = 0.704; preterm delivery, *p* = 0.563), suggesting that IA may influence these outcomes through the mediating mechanism of twin pregnancy rather than directly (Supplementary Tables S21, S24). Additionally, for cesarean section, the direct effect and indirect effect of IA were significant (*p* < 0.05), but the total effect was not significant (*p* = 0.322) (Supplementary Table S25). For PE and placenta accreta, the total effect, direct effect, and indirect effect of IA were all significant (PE: total effect *p* < 0.001, direct effect *p* = 0.011, indirect effect *p* < 0.001; placenta accreta: total effect *p* < 0.001, direct effect *p* < 0.001, indirect effect *p* < 0.001) (Supplementary Tables S21, S23). This indicates that twin pregnancy played a key mediating role in these effects, suggesting that the history of induced abortion may influence the occurrence and development of PE and placenta accreta through its impact on twin pregnancy, thereby revealing its potential risk mechanism in pregnancy health.

**Figure 4:**
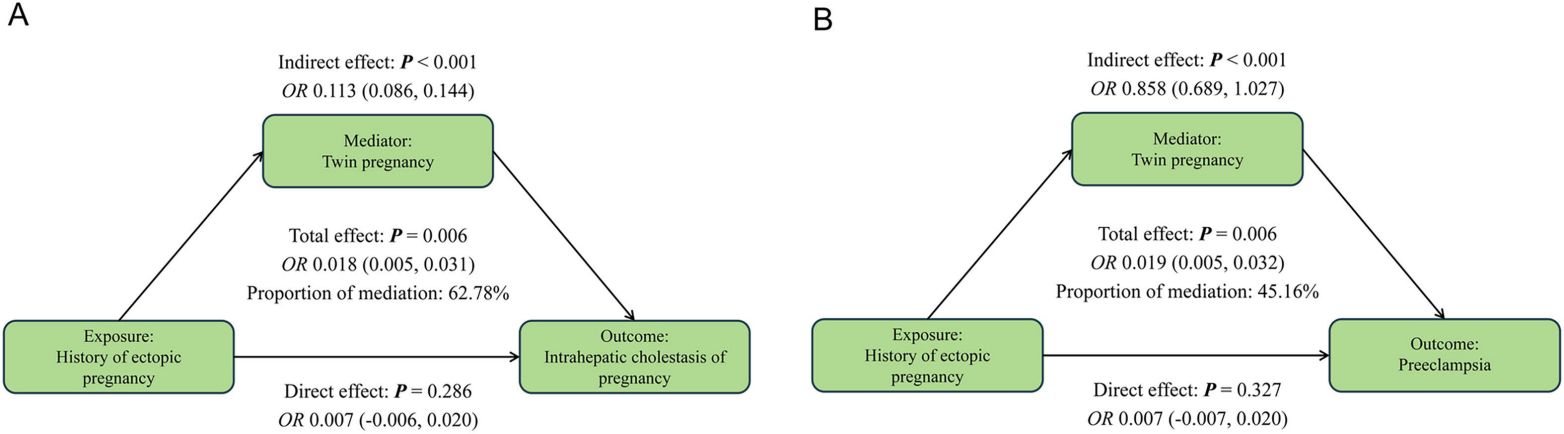
The mediation effects of twin pregnancy on the association of history of ectopic pregnancy with intrahepatic cholestasis of pregnancy (ICP) or preeclampsia (PE) **(A)** ICP; **(B)** PE.

For EP, although the total effect, direct effect, and indirect effect on GDM were not significant (*p* = 0.848; *p* = 0.864; *p* = 0.908) (Supplementary Table S26), the indirect effect on cesarean section was significant (*p* < 0.001) (Supplementary Table S31). However, for PE and ICP, the total effect and indirect effect of EP were significant (*p* < 0.05), while the direct effect was not significant (PE, *p* = 0.327; ICP, *p* = 0.286), indicating that EP may mainly influence these outcomes through the mediating variable of twin pregnancy (Supplementary Tables S27, S30; Figure 4). Moreover, for placenta accreta and preterm delivery, the total effect, direct effect, and indirect effect of EP were all significant (placenta accreta, *p* < 0.001, *p* < 0.001, *p* < 0.001; preterm delivery, *p* < 0.001, *p* = 0.003, *p* < 0.001). These results further emphasize the important mediating role of twin pregnancy in the impact of EP on pregnancy outcomes (Supplementary Tables S29, S30).

## Discussion

This study is the first to identify that the proportion of twin pregnancies significantly increases among women with a history of EP, while it significantly decreases among women with a history of IA. The underlying mechanisms may involve the synergistic effects of multiple factors. Specifically, patients with EP, due to tubal inflammation or anatomical abnormalities, experience a disrupted pelvic microenvironment, which in turn triggers a compensatory hyperreactive ovarian state to enhance ovulation rates (32). Meanwhile, therapeutic salpingectomy enhances ovarian blood flow and follicle recruitment mediated by vascular endothelial growth factor (VEGF). Given the elevated demand for uterine blood flow in twin gestation (33), the impact of salpingectomy may be exacerbated in this population. However, its long-term effects on follicular recruitment and its influence on twin pregnancy risk remain unclear and require further research for validation (34, 35). Additionally, in the EP group, the application rate of ART is significantly higher, and multiple embryo transfer, along with ovulation induction protocols, further amplifies the possibility of twin pregnancies (36). On the other hand, the reduced twin pregnancy rate in the IA group may be related to the following mechanisms: First, mechanical endometrial injury may lead to decreased endometrial receptivity, forcing embryos to implant within a stricter endometrial window. Although such alterations are detrimental to all implantations, the higher demands of twin gestation—requiring two simultaneous successful implantations and greater need for blood supply and endometrial support—mean that even a subtle decline in endometrial quality can significantly inhibit the probability of twin pregnancy (33, 37, 38). Moreover, chronic endometritis may cause an imbalance of Th1/Th2 cytokines and abnormal activity of natural killer (NK) cells. This imbalance may be further exacerbated within the inherently hyperinflammatory and immuno-stressed state of twin pregnancy, which may inhibit the invasive capacity of trophoblasts and the potential for embryonic cleavage (39, 40). Additionally, intrauterine adhesion formation acts as a physical barrier, limiting the implantation space for multiple embryos. Notably, the relatively lower use of ART in the IA group is also an iatrogenic factor contributing to the reduced rate of twin pregnancies, although this factor’s influence was adjusted for in the multivariate logistic regression analysis. Numerous studies have confirmed that adverse obstetric history significantly increases the risk of adverse perinatal outcomes, including GDM, preterm birth, and placental diseases (20, 23). This study further confirms that, regardless of singleton or twin pregnancies, an adverse obstetric history significantly increases the risk of placental diseases. Specifically, in singleton pregnancies, both IA and EP significantly increase the risk of placenta accreta and placenta accreta with bleeding. In twin pregnancies, EP significantly increases the risk of placenta previa and placenta previa with bleeding. This conclusion is consistent with existing research findings (20, 41). For example, as previously shown by W Zhou et al., there is a positive correlation between abortion and placental retention in subsequent singleton live births (42). Additionally, other studies have also suggested that placental abnormalities such as placenta previa, placental retention, and placenta accreta are associated with abortion (43, 44). The mechanism underlying placental abnormalities in subsequent pregnancies after abortion may be related to surgical damage to the endometrium and uterine cavity. In twin gestation, the elevated placental demand readily exceeds the limits of compensation for these injuries, thereby increasing the risk of abnormal placental morphology and function (38, 45, 46). Furthermore, Mélanie Chouinard et al. previously found that women with a history of EP have a 1.21-fold increased risk of placental abruption and a 1.45-fold increased risk of placenta previa, especially in older women (23). This is similar to the current study and may also be due to endometrial damage, inflammatory responses, and angiogenesis abnormalities caused by EP, which can disrupt the decidualization process and thereby reduce placental attachment stability. Additionally, impaired remodeling of uterine spiral arteries and endothelial dysfunction may synergistically exacerbate ischemia and hypoxia of the placental basal membrane. Future research is needed to further analyze the differences in the impact of adverse obstetric history on placental abnormalities in singleton and twin pregnancies.

It should be noted that IA not only significantly increases the risk of FGR and pelvic inflammation in singleton pregnancies but also significantly increases the risk of GDM in twin pregnancies. Induced abortion, as a global public health issue, has far-reaching effects. As of the end of 2017, in 57 countries, especially in developing countries, induced abortion is often completely prohibited or only allowed to save the life of a woman. There have been numerous reports on the adverse perinatal outcomes of subsequent singleton pregnancies following induced abortion (20, 47), indicating that induced abortion can lead to changes in the uterine environment and abnormal immune responses, thereby affecting fetal nutrition supply and maternal resistance to infection. Although few studies have explored the association between induced abortion and GDM, two studies have pointed out that induced abortion is associated with metabolic syndrome or type 2 diabetes. In a cross-sectional survey of 10,375 Chinese women, Xu et al. reported that women with a history of induced abortion were more likely to have metabolic syndrome than those without such a history (9). In another national prospective study, among 302,669 women from the China Kadoorie Biobank, a history of induced abortion was significantly associated with the risk of type 2 diabetes later in life (risk ratio 1.07; 95% CI 1.02–1.12), and this association was related to the number of induced abortions. However, in a singleton pregnancy study from Shanghai, China, it was found that a history of induced abortion did not significantly increase the risk of GDM, which is consistent with this study (48). We further found that a history of induced abortion increased the risk of GDM in twin pregnancies, which may be related to the influence of placental hormones and the additional burden leading to exacerbated insulin resistance in twin pregnancies (49). Interestingly, previous studies have shown that women with a history of SAB have an increased risk of GDM in subsequent pregnancies, but this study did not find that SAB significantly increased the risk, suggesting the need for further research to reveal the underlying mechanisms (48).

Among pregnant women with adverse obstetric history, this study focused on common and clinically significant adverse perinatal outcomes. The results showed that in twin pregnancies with a history of pregnancy loss (including SAB and IA), the risks of GDM, PE, FGR, ICP, placenta accreta with bleeding, and preterm delivery were higher compared with singleton pregnancies (20, 48, 50). Furthermore, the risks in twin pregnancies with IA were not only higher than those in twin pregnancies with SAB but also significantly higher than those in singleton pregnancies with IA. Additionally, twin pregnancies with IA had a 1.64-fold, 3.80-fold, and 2.45-fold increased risk of placenta accreta, placenta previa with bleeding, and pelvic inflammation, respectively. Although previous studies in singleton pregnancies have found that women with a history of SAB have a higher risk of preterm birth and GDM in subsequent pregnancies, no significant association was observed between IA history and related adverse perinatal outcomes. Our study further revealed that women with a history of IA had a higher likelihood of subsequent twin pregnancies. This suggests that clinical examinations should pay more attention to the type of pregnancy loss history and consider whether it is a twin pregnancy. Furthermore, women with twin pregnancies and a history of abortion should be considered a high-risk group for GDM and undergo early screening. Moreover, in twin pregnancies with a history of EP, the risks of PE, ICP, placenta previa with bleeding, preterm delivery, cesarean section, and pelvic inflammation were 3.01, 6.88, 8.47, 6.66, 1.44, and 1.97 times higher, respectively, compared with singleton pregnancies with EP. This phenomenon may be related to the increased maternal physiological burden and placental developmental abnormalities caused by twin pregnancies, leading to insufficient blood supply and enhanced inflammatory responses, which further amplify the impact of EP on related pregnancy complications. Additionally, it must be recognized that while maternal age was statistically adjusted for in this multivariable model, the potential residual confounding effect of this key factor still warrants cautious discussion. Advanced maternal age is a well-established risk factor strongly associated with various obstetric complications, such as preeclampsia, gestational diabetes mellitus, and preterm birth. Recent evidence, exemplified by the study of Yaman FK et al., further confirms the significant impact of maternal age on adverse perinatal outcomes (51). Further investigation may be necessary to clarify the specific influence of age on the observed association. In summary, in the context of adverse obstetric history, twin pregnancies face a higher risk of adverse perinatal outcomes compared with singleton pregnancies. These disparities likely stem from the synergistic effect of multiple mechanisms: the greater placental burden imposed by a twin gestation may amplify the negative impact of pre-existing uterine scarring; its inherently hyper-inflammatory state may have an additive effect with a history of pelvic inflammation; simultaneously, the higher endocrine demand of twin pregnancy could exacerbate the influence of underlying corpus luteum insufficiency (33, 37, 45, 52–54). Based on these findings and mechanistic analyses, risk stratification should be implemented based on the type of adverse obstetric history and the current pregnancy type. Individuals with a medical history of EP may require focused attention on dynamic monitoring of placental function and preparation for multidisciplinary collaboration. Individuals with a medical history of IA may be considered for enhanced early screening for gestational diabetes. For women with a history of SAB, preventive management targeting complications such as preterm labor and preeclampsia is recommended. The exploration of these targeted monitoring protocols may yield novel approaches to the optimization of perinatal management for such high-risk pregnancies. Future research should further explore the specific mechanisms of influence and how to effectively intervene in these risk factors to provide a basis for improving clinical management, which will be an important direction for research.

To analyze the cumulative impact of adverse obstetric histories, pregnant women in this study were categorized according to the number of adverse obstetric history types (1, 2, or 3). The results indicated that significant associations with adverse perinatal outcomes emerged only when two or more types of adverse obstetric histories were present, suggesting a clear cumulative effect. In singleton pregnancies, the presence of two types of adverse obstetric histories increased the risk of outcomes such as placenta previa and placenta accreta with bleeding. This finding is consistent with the analysis by Sun et al., who found that women with a history of SAB and IA had a higher association with the risks of placenta previa and placenta accreta (20). In twin pregnancies, the presence of three types of adverse obstetric histories was associated with a markedly increased risk of GDM and placenta accreta with bleeding. These findings imply that, compared with women who have only a single adverse obstetric history, those with multiple types may require earlier and more frequent monitoring of GDM and placental function. Additionally, among women with twin pregnancies, the coexistence of two adverse obstetric histories was paradoxically associated with a significantly lower risk of cesarean delivery, while the presence of three types showed no significant association. This phenomenon may be explained by a clinical tendency to manage women with two adverse obstetric histories through planned early induction and closely supervised vaginal delivery, thereby reducing cesarean rates (55). In contrast, for women with three types of adverse obstetric histories, the high incidence of severe complications such as placenta accreta often necessitates cesarean delivery (20). Furthermore, the limited sample size in this subgroup may have resulted in insufficient statistical power.

Notably, among women with multiple adverse obstetric histories, twin pregnancies further increased the risks of ICP, placenta accreta with bleeding, and preterm birth compared with singleton pregnancies. This finding suggests that the twin gestational state may amplify the cumulative pathophysiological burden associated with adverse obstetric histories. Therefore, for high-risk women presenting with both twin pregnancies and multiple adverse obstetric histories, early initiation of intensive monitoring of placental function and metabolic indicators is strongly recommended.

Mediation analysis revealed the complex mechanisms between adverse obstetric history and adverse perinatal outcomes, clarifying the key role of twin pregnancy as a mediating variable. The results showed that SAB had significant total and direct effects on GDM (48), but its indirect effect was not statistically significant. This suggests that SAB may directly increase the risk of GDM through abnormal endometrial repair or metabolic pathways (such as persistent insulin resistance or chronic inflammation directly impairing glucose homeostasis), rather than through twin pregnancy as a mediator. Additionally, the mediation effects of SAB on other adverse perinatal outcomes were not significant, indicating that its pathogenic mechanisms may be independent of the pathophysiological processes of twin pregnancy. In contrast, IA had significant indirect effects on ICP and preterm delivery, while its direct effects were not significant. This indicates that twin pregnancy is a core mediating pathway through which IA affects these complications. Notably, IA had significant total, direct, and indirect effects on PE and placenta accreta, suggesting dual pathogenic mechanisms. These mechanisms may involve both twin pregnancy-mediated placental dysfunction and direct exacerbation of PE and placenta accreta risks through immune dysregulation or endothelial injury (47, 56). Therefore, when optimizing obstetric care for pregnant women with twin pregnancies, the above-mentioned factors need to be comprehensively considered to more specifically reduce the risk of placental dysfunction and related complications (57). These findings suggest that for pregnant women with IA history, upon confirmation of pregnancy type in the first trimester, those with a twin gestation should consider prophylactic measures for PE (e.g., low-dose aspirin) and enhance surveillance and early management of ICP. For women with EP history, increased monitoring of cervical length and assessment of preterm birth risk, along with advanced planning for delivery strategies, may be warranted. Moreover, regarding cesarean section, IA had a significant indirect effect, but its overall effect was not significant. This suggests that twin pregnancy may indirectly influence the need for cesarean section through specific clinical pathways (such as fetal malposition or labor dystocia), rather than through an overall effect. Finally, for EP, its effects on PE and ICP were mainly mediated through twin pregnancy, while its effects on placenta accreta and preterm delivery involved both direct and indirect pathways. This may be related to tubal damage and chronic pelvic inflammation caused by EP (58, 59). This pathological state not only directly impairs the endometrial–placental interface but may also indirectly promote complications through abnormal placental perfusion in twin pregnancies. Additionally, the significant indirect effect of EP-related cesarean section risk further supports twin pregnancy as an important mediating factor in clinical decision-making. This reinforces our understanding of the mechanisms through which twin pregnancy influences adverse perinatal outcomes in the context of adverse obstetric history and provides a new perspective for clinical management. It may become a key indicator for predicting and influencing adverse obstetric and perinatal outcomes in women with twin pregnancies. Furthermore, the results of the mediation analysis suggest that the pathways through which SAB, IA, and EP influence perinatal outcomes may differ, potentially stemming from distinct underlying pathological mechanisms. SAB is predominantly associated with intrinsic factors, such as embryonic chromosomal abnormalities or maternal endocrine conditions (60). As indicated by the available research, IA and EP result in more pronounced anatomical and inflammatory damage due to damage to the basal layer of the endometrium (45). The condition known as pelvic inflammatory disease has been observed to be associated with tubal pathology (61). This mechanistic divergence may render IA and EP more susceptible to influencing perinatal outcomes via the intermediary pathway of twin pregnancy, whereas SAB’s effects may be realized through alternative pathways. Furthermore, SAB itself exhibits considerable heterogeneity (20, 41), and its overall effect may be partially obscured without stratified analysis. The specific mechanisms underlying this mediating pathway require further investigation.

This study shows that prior ectopic pregnancy increases, while prior induced abortion decreases, the likelihood of twin pregnancy. Adverse obstetric histories—particularly induced abortion and ectopic pregnancy—elevate risks of adverse perinatal outcomes, and twin pregnancy amplifies these risks. Cumulative effects are seen with multiple adverse history types, and twin pregnancy partially mediates the effects of induced abortion and ectopic pregnancy on certain outcomes. Clinically, risk stratification by history type and pregnancy type is essential: enhanced placental monitoring for prior ectopic pregnancy with twins, early GDM screening for prior induced abortion, and intensive surveillance for recurrent adverse histories. These findings inform tailored antenatal care to reduce complications in this vulnerable population.

## Strengths and limitations

The strengths of our study include the utilization of a multi-year database from a tertiary teaching hospital, which not only ensures the richness and continuity of the study data but also provides a foundation enhancing the depth and breadth of the research. Our cases were derived from strict pathological or clinical diagnoses, rather than relying solely on ICD codes, which largely ensures the accuracy of the diagnoses of the study population. At the analytical level, this study not only examined individual types of adverse obstetric histories but also explored the cumulative effects of multiple adverse obstetric events on perinatal outcomes. Such a design better reflects the complexity of real-world clinical scenarios and enhances the practical applicability of the conclusions. Moreover, the same medical team managed the patients in the same center, employing standardized surgical strategies and preoperative, intraoperative, and postoperative management protocols. This consistency reduces potential outcome biases that may arise from different caregivers. Additionally, the study meticulously documented common pregnancy comorbidities and placental-related information, including GDM, PE, ICP, FGR, preterm delivery, placenta accreta, and placenta previa.

However, the study has the following limitations: First, this was a single-center retrospective cohort study, which inherently carries risks of selection bias and information bias. Given the retrospective design, adverse obstetric history was primarily obtained by maternal self-report; we attempted medical-record verification where available, but verification was incomplete. Consequently, recall bias and misclassification remain possible—particularly for SA, IA, or EP managed entirely outside our institution, as those events were not captured in our electronic medical records. These limitations may introduce both selection bias (since women with events managed elsewhere might differ systematically from those whose events were recorded at our hospital) and information bias (due to inaccurate recall or incomplete documentation). Any residual non-differential misclassification would be expected to bias associations toward the null, though differential recall cannot be excluded and might attenuate or exaggerate specific estimates. This design-related limitation should be considered when interpreting the magnitude of our findings. Second, existing literature indicates that the interpregnancy interval following abortion may affect the occurrence of perinatal complications, but this study failed to analyze the impact of this factor on outcomes (62). We acknowledge that the interval between the adverse obstetric event and the subsequent pregnancy could influence the risk of adverse perinatal outcomes, and its omission from our analysis may introduce residual confounding. Unfortunately, our dataset did not include the dates of prior abortions or ectopic pregnancies, preventing us from calculating the interpregnancy interval or adjusting for it in the regression models. Third, our EMR data indicated only the presence of each type of adverse obstetric history but not the number of occurrences. Thus, we could not examine whether recurrent events (e.g., multiple SABs) confer a different risk compared with a single event. This is an important limitation, as the cumulative effect of repeated adverse obstetric events may influence subsequent perinatal outcomes. Another significant limitation is the absence of data on chorionicity (monochorionic versus dichorionic twins), a major determinant of outcomes in twin pregnancies. The inability to stratify our analysis by chorionicity may have masked important differences in risk. Furthermore, this limitation restricts our ability to fully assess the associations between prior adverse obstetric histories and the subsequent rate of twin pregnancy, given the distinct physiological mechanisms underlying monochorionic and dichorionic twin gestations. However, given that our primary focus was on comparing twin versus singleton pregnancies rather than chorionicity-specific effects within twins, this limitation is unlikely to substantially alter our main conclusions, though it should be noted as a potential source of residual confounding. Additionally, although we adjusted for ART in our regression models, we lacked detailed ART-related information, such as the number of embryos transferred, ovulation induction protocols, or fresh versus frozen embryo transfer status. Twin pregnancies are frequently conceived via ART, which is itself associated with increased risks of several maternal and perinatal complications. Some studies report that ART-conceived DCDA twins have poorer outcomes, which further underscores the importance of capturing granular ART data, as unmeasured ART factors (e.g., embryo transfer number or cycle characteristics) may confound the associations we observed. Therefore, residual confounding due to unmeasured ART factors cannot be excluded and may have biased our estimates (63). Additionally, we lacked data on prior uterine surgeries (e.g., myomectomy or hysteroscopic procedures), which are known risk factors for placental abnormalities and may have contributed to residual confounding. Moreover, in analyses concerning induced abortions, due to missing data, we were unable to further distinguish between medical and surgical abortions, nor did we obtain specific details such as gestational age at the time of termination or the method employed. These factors have been proven to have a strong association with subsequent perinatal outcomes, and the presence of unobserved confounding factors has the potential to introduce bias into the risk estimates (47, 64–66). Finally, over time, methotrexate has become increasingly common as a drug treatment, especially among women who have previously undergone treatment for ectopic pregnancy (67). It has been reported that from 2006 to 2015, the use of methotrexate nearly doubled, and most ectopic pregnancies require medical intervention (6, 67). Different treatment modalities may play a role in the association pathway between ectopic pregnancy and future adverse outcomes, but this study lacked such information, which may affect the reliability of the conclusions (23).

## Data Availability

The raw data supporting the conclusions of this article will be made available by the authors, without undue reservation.
