# Supplementary material for: Association between adverse obstetric history and perinatal outcomes in singleton and twin pregnancies: a retrospective study
Source: Front Med (Lausanne). 2026 Jun 30;13:1814198. doi: 10.3389/fmed.2026.1814198 (PMC13371165; doi:10.3389/fmed.2026.1814198)
Supplement: Supplementary file 1 [file Supplementary_file_1.docx]

**Association between Adverse Obstetric History and Perinatal Outcomes in Singleton and Twin Pregnancies: A Retrospective Study**

Wei-Zhen Tang ^a, b¶^, Xia Li ^b, c¶^, Hong-Yu Xu ^a, b^, Qin-Yu Cai ^a, b^ , Ni-Ya Zhou ^a, d^, Yi-Fan Zhao ^b^，Hao-Wen Chen ^b,c^, Yue-Tang ^b^, Fei Han ^b, c,^*, Tai-Hang Liu ^b, c,^*，Kai Ye ^a,d,^*

**Affiliations:**

^a^ Department of Obstetrics and Gynecology，Women and Children’s Hospital of Chongqing Medical University, Chongqing 401147, China.

^b^ School of Basic Medical Sciences, Chongqing Medical University, Chongqing 400016, China.

^c^ The Joint International Research Laboratory of Reproduction and Development, Chongqing Medical University, Chongqing, 400016, China.

^d^ Department of Obstetrics and Gynecology, Chongqing Health Center for Women and Children 401147, China.

***Correspondence:**

* Fei Han (han16897723@163.com); Tai-Hang Liu [(liuth@cqmu.edu.cn);](mailto:(liuth@cqmu.edu.cn);) Kai Ye (446702241@qq.com). Box 197, Chongqing Medical University, No.1 Yixueyuan Rd, Chongqing, 400016, PR China. Tel.: +86 023 68485868.

¶ W.Z. Tang and X. Li contributed equally to this work.

**Appendix 1. Rationale for Selection of Adverse Perinatal Outcome Indicators**

**Placental abnormalities (placenta previa, placenta accreta spectrum, and associated hemorrhage)**

Induced abortion, cesarean section, and surgery for ectopic pregnancy can all damage the endometrium and disrupt the intrauterine microenvironment, leading to abnormal placental implantation. Placental abnormalities are major causes of postpartum hemorrhage and perinatal mortality, carrying extremely high maternal and fetal safety risks; therefore, they have been included as core monitoring indicators.

**Gestational diabetes mellitus (GDM), preeclampsia (PE), fetal growth restriction (FGR), and intrahepatic cholestasis of pregnancy (ICP)**

Endometrial damage resulting from prior adverse pregnancy outcomes can impair endometrial receptivity, induce chronic low-grade inflammation, and cause placental dysfunction, thereby increasing the risk of GDM, PE, FGR, and ICP. These complications represent the most common disorders during pregnancy and significantly elevate the risk of adverse maternal and neonatal outcomes in both the short and long term, warranting important monitoring value.

**Preterm birth**

The number of induced abortions demonstrates a dose-response relationship with the risk of subsequent preterm birth, and a history of ectopic pregnancy is also an independent risk factor for preterm delivery. Preterm birth is the leading cause of neonatal mortality and long-term adverse prognosis globally, imposing a substantial public health burden; thus, it must be incorporated into outcome assessment.

**Cesarean section**

Surgical abortion and surgery for ectopic pregnancy can cause pelvic adhesions and uterine scarring, which not only directly increase the technical need for subsequent cesarean delivery but are also closely associated with maternal and neonatal complications in the near and long term. Therefore, cesarean section serves as both a potential consequence of prior surgical exposure and a direct manifestation of adverse outcomes.

**Pelvic inflammatory disease**

Pelvic inflammatory disease is an important sequela of reproductive tract infection and is closely associated with ectopic pregnancy, infertility, and adverse pregnancy outcomes. Its inclusion in the indicator system allows indirect reflection of prior reproductive tract infection status and its long-term reproductive health damage.

**Table S1.** Demographic and clinical characteristics of women with history of adverse pregnancy.

| **Characteristic [N (%)]** | **SAB** | **Non-SAB** | **Statistic (χ2)** | ***P*.value** | **IA** | **Non-IA** | **Statistic (χ2)** | ***P*.value** | **EP** | **Non-EP** | **Statistic (χ2)** | ***P*.value** |
| --- | --- | --- | --- | --- | --- | --- | --- | --- | --- | --- | --- | --- |
| Age (Years) | 31.58[28.67,34.64] | 30.51[27.59,33.78] | -8.682 | <0.001* | 31.89[28.39,34.85] | 29.89[27.27,32.96] | -23.432 | <0.001* | 32.47[29.72,34.93] | 30.493[27.57,33.75] | -13.120 | <0.001* |
| PBMI, M (Q₁, Q₃) | 21.60[19.63,23.83] | 21.23[19.13,23.71] | -4.473 | <0.001* | 21.41[19.40,23.88] | 21.09[18.90,23.56] | -6.649 | <0.001* | 21.65[19.88,23.61] | 21.22[19.11,23.73] | -4.862 | <0.001* |
| ART, n(%) | 60(3.93) | 541(3.46) | 0.933 | 0.334 | 168(2.35) | 433(4.32) | 48.017 | <0.001* | 118(10.16) | 483(3.02) | 163.865 | <0.001* |
| Nulliparity, n (%) | 964(63.17) | 1030(65.81) | 4.290 | 0.038* | 3511(49.08) | 7755(77.35) | 1478.240 | <0.001* | 797(68.65) | 10469(65.35) | 5.204 | 0.023* |
| Age of first menstruation | 12.00[11.00,13.00] | 12.00[11.00,13.00] | -1.769 | 0.063 | 12.00[11.00,13.00] | 12.00[11.00,13.00] | 0.843 | 0.376 | 12.00[11.00,13.00] | 12.00[11.00,13.00] | -1.797 | 0.059 |
| Menstrual cycle | 28.00[28.00,30.00] | 28.00[28.00,29.00] | -0.610 | 0.497 | 28.00[28.00,28.00] | 28.00[28.00,30.00] | 5.477 | <0.001* | 28.00[28.00,28.00] | 28.00[28.00,28.00] | 2.761 | 0.002* |
| Menstrual duration | 5.00[4.00,6.00] | 5.00[4.00,6.00] | 2.121 | 0.029* | 5.00[4.00,5.00] | 5.00[4.00,5.00] | 17.369 | <0.001* | 5.00[4.00,6.00] | 5.00[4.00,6.00] | 2.114 | 0.029 |
| Endocrine system diseases | 438(28.70) | 4427(28.28) | 0.122 | 0.727 | 1854(25.92) | 3011(30.03) | 34.850 | <0.001* | 257(22.14) | 4608(28.77) | 23.441 | <0.001* |
| History of smoking, n(%) | 32(2.11) | 429(2.76) | 2.206 | 0.138 | 315(4.44) | 146(1.47) | 139.362 | <0.001* | 34(2.95) | 427(2.69) | 0.290 | 0.590 |
| History of alcoholism, n(%) | 256(16.92) | 2423(15.70) | 1.538 | 0.215 | 1309(18.70) | 1370(13.78) | 74.722 | <0.001* | 202(17.69) | 2477(15.67) | 3.246 | 0.072 |

**Abbreviation:** SAB, Spontaneous Abortion History; IA, Induced Abortion History; EP, Ectopic Pregnancy History; PBMI, Pre-pregnancy body mass Index; ART, Assisted reproductive technology

*p < 0.05

**Table S2.** The demographic and clinical characteristics of SAB in women with twin and singleton pregnancies.

| **Characteristic [N (%)]** | **Twin pregnancy** | | | | **Singleton pregnancy** | | | |
| --- | --- | --- | --- | --- | --- | --- | --- | --- |
|  | **SAB** | **Non-SAB** | **Statistic (χ2)** | ***P*.value** | **SAB** | **Non-SAB** | **Statistic (χ2)** | ***P*.value** |
| Age (Years) | 31.35[28.91,34.21] | 30.93[28.61,33.44] | -1.507 | 0.132 | 31.62[28.67,34.67] | 30.47[27.51,33.82] | -8.605 | <0.001* |
| PBMI, M (Q₁, Q₃) | 21.64[20.14,23.53] | 21.50[20.03,23.61] | -0.605 | 0.546 | 21.57[19.56,23.92] | 21.19[19.04,23.73] | -4.500 | <0.001* |
| ART, n(%) | 25(20.49) | 273(21.84) | 0.119 | 0.730 | 35(2.49) | 268(1.86) | 2.720 | 0.099 |
| Primigravida, n (%) | 100(81.97) | 1085(86.80) | 2.205 | 0.138 | 864(61.54) | 9217(63.99) | 3.326 | 0.068 |
| Age of first menstruation | 12.00[11.00,13.00] | 12.00[11.00,13.00] | -1.575 | 0.100 | 12.00[11.00,13.00] | 12.00[11.00,13.00] | -1.380 | 0.147 |
| Menstrual cycle | 28.00[28.00,29.00] | 28.00[28.00,28.00] | -1.515 | 0.089 | 28.00[28.00,30.00] | 28.00[28.00,29.00] | -0.200 | 0.824 |
| Menstrual duration | 7.00[5.00,7.00] | 6.00[5.00,7.00] | -1.498 | 0.113 | 5.00[4.00,6.00] | 5.00[4.00,6.00] | 2.498 | 0.010* |
| Endocrine system diseases | 31(25.41) | 275(22.00) | 0.746 | 0.388 | 407(28.99) | 4152(28.83) | 0.017 | 0.897 |
| History of smoking, n(%) | 2(1.64) | 24(1.93) | 0.050 | 0.823 | 30(2.15) | 405(2.83) | 2.172 | 0.141 |
| History of alcoholism, n(%) | 27(22.50) | 159(12.84) | 8.630 | 0.003* | 229(16.44) | 2264(15.95) | 0.226 | 0.635 |

**Abbreviation:** SAB, Spontaneous Abortion History; IA, Induced Abortion History; EP, Ectopic Pregnancy History; PBMI, Pre-pregnancy body mass Index; ART, Assisted reproductive technology.

*p < 0.05

**Table S3.** The demographic and clinical characteristics of IA in women with twin and singleton pregnancies.

| **Characteristic [N (%)]** | **Twin pregnancy** | | | | **Singleton pregnancy** | | | |
| --- | --- | --- | --- | --- | --- | --- | --- | --- |
|  | **IA** | **Non-IA** | **Statistic (χ2)** | ***P*.value** | **IA** | **Non-IA** | **Statistic (χ2)** | ***P*.value** |
| Age (Years) | 31.36[28.61,34.34] | 30.89[28.60,33.26] | -3.284 | 0.001* | 31.90[28.37,34.87] | 29.72[27.15,32.89] | -23.553 | <0.001* |
| PBMI, M (Q₁, Q₃) | 21.63[20.03,23.53] | 21.48[20.06,23.61] | -0.268 | 0.789 | 21.37[19.38,23.92] | 21.03[18.75,23.56] | -7.253 | <0.001* |
| ART, n(%) | 69(19.06) | 229(22.67) | 2.045 | 0.153 | 99(1.46) | 204(2.26) | 13.354 | <0.001* |
| Primigravida, n (%) | 260(71.82) | 925(91.58) | 88.397 | <0.001* | 3251(47.87) | 6830(75.75) | 1304.146 | <0.001* |
| Age of first menstruation | 12.00[11.00,13.00] | 12.00[11.00,13.00] | -1.803 | 0.060 | 12.00[11.00,13.00] | 12.00[11.00,13.00] | 1.210 | 0.204 |
| Menstrual cycle | 28.00[28.00,28.00] | 28.00[28.00,28.00] | 1.882 | 0.034* | 28.00[28.00,29.00] | 28.00[28.00,30.00] | 5.388 | <0.001* |
| Menstrual duration | 6.00[5.00,7.00] | 7.00[5.00,7.00] | 3.406 | <0.001* | 5.00[3.00,5.00] | 5.00[4.00,6.00] | 16.881 | <0.001* |
| Endocrine system diseases | 85(23.48) | 221(21.88) | 0.393 | 0.531 | 1769(26.05) | 2790(30.95) | 45.314 | <0.001* |
| History of smoking, n(%) | 12(3.32) | 14(1.39) | 5.304 | 0.021* | 303(4.50) | 132(1.47) | 130.544 | <0.001* |
| History of alcoholism, n(%) | 56(15.73) | 130(12.97) | 1.688 | 0.194 | 1253(18.86) | 1240(13.87) | 70.623 | <0.001* |

**Abbreviation:** SAB, Spontaneous Abortion History; IA, Induced Abortion History; EP, Ectopic Pregnancy History; PBMI, Pre-pregnancy body mass Index; ART, Assisted reproductive technology.

*p < 0.05

**Table S4.** The demographic and clinical characteristics of EP in women with twin and singleton pregnancies.

| **Characteristic [N (%)]** | **Twin pregnancy** | | | | **Singleton pregnancy** | | | |
| --- | --- | --- | --- | --- | --- | --- | --- | --- |
|  | **EP** | **Non-EP** | **Statistic (χ2)** | ***P*.value** | **EP** | **Non-EP** | **Statistic (χ2)** | ***P*.value** |
| Age (Years) | 31.85[29.44,34.21] | 30.90[28.48,33.36] | -2.858 | 0.004* | 32.59[29.73,35.04] | 30.45[27.50,33.784 | -12.784 | <0.001* |
| PBMI, M (Q₁, Q₃) | 21.87[20.06,23.63] | 21.48[20.06,23.53] | -0.833 | 0.405 | 21.64[19.81,23.60] | 21.12[19.03,23.73] | -4.581 | <0.001* |
| ART, n(%) | 54(29.03) | 244(20.57) | 6.766 | 0.009* | 64(6.56) | 239(1.61) | 119.372 | <0.001* |
| Primigravida, n (%) | 152(81.72) | 1033(87.10) | 3.952 | 0.047* | 645(66.15) | 9436(63.62) | 2.553 | 0.110 |
| Age of first menstruation | 12.00[11.00,13.00] | 12.00[11.00,13.00] | -1.029 | 0.282 | 12.00[11.00,13.00] | 12.00[11.00,13.00] | -1.404 | 0.140 |
| Menstrual cycle | 28.00[28.00,28.00] | 28.00[28.00,28.00] | 0.683 | 0.442 | 28.00[28.00,28.00] | 28.00[28.00,29.00] | 2.528 | 0.005* |
| Menstrual duration | 7.00[5.00,7.00] | 6.00[5.00,7.00] | 0.010 | 0.992 | 5.00[4.00,6.00] | 5.00[4.00,6.00] | 2.205 | 0.023* |
| Endocrine system diseases | 38(20.43) | 268(22.60) | 0.436 | 0.509 | 219(22.46) | 4340(29.26) | 20.598 | <0.001* |
| History of smoking, n(%) | 4(2.19) | 22(1.86) | 0.090 | 0.764 | 30(3.10) | 405(2.75) | 0.401 | 0.527 |
| History of alcoholism, n(%) | 44(24.18) | 142(12.08) | 19.525 | <0.001* | 158(16.46) | 2335(15.96) | 0.164 | 0.685 |

**Abbreviation:** SAB, Spontaneous Abortion History; IA, Induced Abortion History; EP, Ectopic Pregnancy History; PBMI, Pre-pregnancy body mass Index; ART, Assisted reproductive technology.

*p < 0.05

**Table S5.** The impact of adverse obstetric history on the risk of adverse perinatal outcomes in twin versus singleton pregnancies.

|  | **Twin pregnancy** | | | | | | | | | **Singleton pregnancy** | | | | | | | | |
| --- | --- | --- | --- | --- | --- | --- | --- | --- | --- | --- | --- | --- | --- | --- | --- | --- | --- | --- |
|  | **Non-SAB** | **SAB[aOR^a^(95%CI)]** | **p** | **Non-IA** | **IA[aOR(95%CI)]** | **p** | **Non-EP** | **EP[aOR(95%CI)]** | **p** | **Non-SAB** | **SAB[aOR(95%CI)]** | **p** | **Non-IA** | **IA[aOR(95%CI)]** | **p** | **Non-EP** | **EP[aOR(95%CI)]** | **p** |
| GDM | Reference | 1.23(0.78,1.93) | 0.361 | Reference | 1.47(1.03,2.11) | 0.034* | Reference | 1.23(0.83,1.82) | 0.300 | Reference | 0.98(0.88,1.10) | 0.788 | Reference | 0.86(0.78,0.93) | <0.001* | Reference | 0.95(0.82,1.09) | 0.443 |
| PE | Reference | 0.85(0.49,1.43) | 0.557 | Reference | 0.90(0.65,1.47) | 0.901 | Reference | 0.92(0.58,1.45) | 0.737 | Reference | 1.10(0.84,1.42) | 0.475 | Reference | 0.95(0.77,1.21) | 0.619 | Reference | 1.16(0.85,1.55) | 0.323 |
| FGR | Reference | 1.20(0.50,2.60) | 0.444 | Reference | 1.19(0.62,2.35) | 0.601 | Reference | 0.26(0.08,0.66) | 0.012* | Reference | 1.30(0.83,1.94) | 0.227 | Reference | 0.97(0.68,1.40) | 0.864 | Reference | 1.87(1.19,2.81) | 0.004* |
| ICP | Reference | 0.60(0.32,1.05) | 0.089 | Reference | 1.32(0.86,2.03) | 0.203 | Reference | 1.37(0.86,2.15) | 0.174 | Reference | 0.92(0.67,1.22) | 0.564 | Reference | 1.18(0.94,1.49) | 0.151 | Reference | 1.10(0.77,1.51) | 0.597 |
| Placenta accreta | Reference | 0.82(0.45,1.43) | 0.492 | Reference | 1.18(0.76,1.84) | 0.457 | Reference | 1.10(0.68,1.75) | 0.700 | Reference | 1.16(0.95,1.40) | 0.139 | Reference | 1.29(1.12,1.50) | 0.001* | Reference | 1.76(1.44,2.13) | <0.001* |
| Placenta accreta with bleeding | Reference | 1.34(0.47,3.40) | 0.555 | Reference | 1.38(0.59,3.39) | 0.465 | Reference | 1.44(0.58,3.43) | 0.412 | Reference | 0.44(0.20,0.84) | 0.023* | Reference | 1.62(1.19,2.21) | 0.002* | Reference | 1.88(1.16,2.92) | 0.007* |
| Placenta previa | Reference | 0.44(0.07,1.58) | 0.282 | Reference | 2.58(0.98,8.05) | 0.072 | Reference | 4.59(1.84,12.06) | 0.001* | Reference | 1.32(0.96,1.78) | 0.078 | Reference | 1.19(0.96,1.47) | 0.120 | Reference | 1.40(0.97,1.95) | 0.059 |
| Placenta previa with bleeding | Reference | 0.62(0.09,2.37) | 0.538 | Reference | 2.20(0.71,8.21) | 0.194 | Reference | 5.71(1.90,19.19) | 0.003* | Reference | 1.06(0.55,1.85) | 0.863 | Reference | 1.37(0.93,2.01) | 0.113 | Reference | 1.16(0.54,2.18) | 0.669 |
| Premature delivery | Reference | 1.27(0.80,1.97) | 0.303 | Reference | 0.78(0.55,1.12) | 0.181 | Reference | 1.31(0.89,1.94) | 0.171 | Reference | 0.95(0.72,1.23) | 0.698 | Reference | 1.07(0.91,1.25) | 0.417 | Reference | 1.30(0.98,1.71) | 0.064 |
| Cesarean section | Reference | 0.99(0.64,1.53) | 0.959 | Reference | 0.58(0.41,0.81) | 0.002* | Reference | 0.85(0.58,1.24) | 0.392 | Reference | 0.98(0.88,1.10) | 0.788 | Reference | 0.95(0.87,1.03) | 0.219 | Reference | 1.09(0.95,1.24) | 0.229 |
| Pelvic inflammation | Reference | 0.63(0.32,1.14) | 0.144 | Reference | 0.87(0.57,1.34) | 0.531 | Reference | 1.43(0.90,2.25) | 0.128 | Reference | 1.06(0.84,1.32) | 0.631 | Reference | 0.89(0.76,1.02) | 0.101* | Reference | 1.69(1.34,2.10) | <0.001* |

**Abbreviation:** SAB, Spontaneous Abortion History; IA, Induced Abortion History; EP, Ectopic Pregnancy History; GDM: Gestational Diabetes Mellitus; PE: Preeclampsia; FGR: Fetal Growth Restriction; ICP: Intrahepatic Cholestasis of Pregnancy.

^a.^Adjusting for factors including maternal age, PBMI, ART, nulliparity, age of first menstruation, menstrual cycle, menstrual duration, endocrine system diseases, history of smoking, and history of alcoholism.

*p < 0.05

**Table S6.** The impact of adverse obstetric history on the risk of adverse perinatal outcomes in twin versus singleton pregnancies.

|  | **Twin pregnancy** | | | | | | | | | **Singleton pregnancy** | | | | | | | | |
| --- | --- | --- | --- | --- | --- | --- | --- | --- | --- | --- | --- | --- | --- | --- | --- | --- | --- | --- |
|  | **SAB** | **Non-SAB** | **p** | **IA** | **Non-IA** | **p** | **EP** | **Non-EP** | **p** | **SAB** | **Non-SAB** | **p** | **IA** | **Non-IA** | **p** | **EP** | **Non-EP** | **p** |
| GDM, n(%) | 56(45.90) | 448(35.84) | 0.028* | 159(43.923) | 345(34.16) | <0.001* | 79(42.47) | 425(35.84) | 0.081 | 581(41.38) | 5144(35.71) | <0.001* | 2405(35.41) | 3320(36.82) | 0.067 | 373(38.256) | 5352(36.082) | 0.171 |
| PE, n(%) | 20(16.39) | 233(18.64) | 0.541 | 63(17.40) | 190(18.81) | 0.553 | 31(16.67) | 222(18.72) | 0.502 | 69(4.92) | 571(3.96) | 0.085 | 259(3.81) | 381(4.23) | 0.193 | 53(5.44) | 587(3.96) | 0.023* |
| FGR, n(%) | 8(6.56) | 101(8.08) | 0.553 | 24(6.63) | 85(8.42) | 0.281 | 6(3.23) | 103(8.69) | 0.010* | 25(1.78) | 200(1.39) | 0.236 | 83(1.22) | 142(1.58) | 0.064 | 25(2.56) | 200(1.35) | 0.002* |
| ICP, n(%) | 15(12.30) | 218(17.44) | 0.149 | 65(17.96) | 168(16.63) | 0.565 | 36(19.36) | 197(16.61) | 0.354 | 48(3.42) | 567(3.94) | 0.338 | 247(3.64) | 368(4.08) | 0.152 | 39(4.00) | 576(3.88) | 0.855 |
| Placenta accreta, n(%) | 20(16.39) | 159(12.72) | 0.250 | 62(17.13) | 117(11.58) | 0.007 | 40(21.51) | 185(15.60) | 0.043* | 132(9.40) | 1104(7.67) | 0.021* | 681(10.03) | 555(6.16) | <0.001* | 136(13.95) | 1100(7.42) | <0.001* |
| Placenta accreta with bleeding | 6(4.92) | 37(2.96) | 0.236 | 14(3.87) | 29(2.87) | 0.351 | 9(4.84) | 34(2.87) | 0.151 | 8(0.57) | 180(1.25) | 0.025* | 102(1.50) | 86(0.95) | 0.002* | 22(2.26) | 166(1.12) | 0.002* |
| Placenta previa, n(%) | 2(1.64) | 42(3.36) | 0.303 | 15(4.14) | 29(2.87) | 0.238 | 12(6.45) | 32(2.70) | 0.007* | 48(3.42) | 351(2.44) | 0.025* | 200(2.95) | 199(2.21) | 0.003* | 38(3.90) | 361(2.43) | 0.005* |
| Placenta previa with bleeding, n(%) | 2(1.64) | 20(1.60) | 0.974 | 10(2.76) | 12(1.19) | 0.041* | 9(4.84) | 13(1.10) | <0.001* | 12(0.86) | 111(0.77) | 0.732 | 66(0.97) | 57(0.63) | 0.016* | 9(0.92) | 114(0.77) | 0.595 |
| Premature delivery | 36(29.51) | 309(24.72) | 0.245 | 94(25.97) | 251(24.85) | 0.675 | 58(31.18) | 287(24.20) | 0.041* | 63(4.49) | 657(4.56) | 0.899 | 323(4.76) | 397(4.40) | 0.293 | 58(5.95) | 662(4.46) | 0.031* |
| Cesarean section | 77(63.12) | 772(61.76) | 0.769 | 208(57.46) | 641(63.47) | 0.043* | 117(62.90) | 732(61.720) | 0.757 | 661(47.08) | 6521(45.27) | 0.194 | 3310(48.73) | 3872(42.95) | <0.001* | 491(50.36) | 6691(45.11) | 0.001* |
| Pelvic inflammation | 13(10.66) | 212(16.96) | 0.073 | 59(16.30) | 166(16.44) | 0.952 | 40(21.51) | 185(15.60) | 0.043* | 91(6.48) | 808(5.61) | 0.178 | 399(5.88) | 500(5.55) | 0.377 | 100(10.26) | 799(5.39) | <0.001* |

**Abbreviation:** SAB, Spontaneous Abortion History; IA, Induced Abortion History; EP, Ectopic Pregnancy History; GDM: Gestational Diabetes Mellitus; PE: Preeclampsia; FGR: Fetal Growth Restriction; ICP: Intrahepatic Cholestasis of Pregnancy

*p < 0.05

**Table S7.** Comparison of adverse perinatal outcomes associated with twin and singleton pregnancies in patients with adverse obstetric history.

|  | **SAB** |  |  |  | **IA** |  |  |  | **EP** |  |  |  |
| --- | --- | --- | --- | --- | --- | --- | --- | --- | --- | --- | --- | --- |
|  | **Twin pregnancy** | **Singleton pregnancy** | **p.value** | **aOR^a^(95%CI)** | **Twin pregnancy** | **Singleton pregnancy** | **p.value** | **aOR(95%CI)** | **Twin pregnancy** | **Singleton pregnancy** | **p.value** | **aOR(95%CI)** |
| GDM, n(%) | 56(45.90) | 581(41.38) | 0.332 | 1.11(1.08,1.14) | 159(43.92) | 2405(35.41) | <0.001* | 1.474(1.17,1.85) | 79(42.47) | 373(38.26) | 0.280 | 1.22(0.86,1.73) |
| PE, n(%) | 20(16.39) | 69(4.92) | <0.001* | 3.05(1.69,5.30) | 63(17.40) | 259(3.81) | <0.001* | 5.35(3.85,7.34) | 31(16.67) | 53(5.44) | <0.001* | 3.01(1.80,4.97) |
| FGR, n(%) | 8(6.56) | 25(1.78) | <0.001* | 3.08(1.22,7.04) | 24(6.63) | 83(1.22) | <0.001* | 5.87(3.51,9.46) | 6(3.23) | 25(2.56) | 0.608 | 1.29(0.46,3.09) |
| ICP, n(%) | 15(12.30) | 48(3.42) | <0.001* | 4.40(2.24,8.24) | 65(17.96) | 247(3.64) | <0.001* | 6.35(4.60,8.66) | 36(19.36) | 39(4.00) | <0.001* | 6.88(4.13,11.46) |
| Placenta accreta, n(%) | 20(16.39) | 132(9.40) | 0.013* | 1.18(0.63,2.09) | 62(17.13) | 681(10.03) | <0.001* | 1.64(1.20,2.21) | 34(18.28) | 136(13.95) | 0.126 | 1.25(0.80,1.91) |
| Placenta accreta with bleeding, n(%) | 6(4.92) | 8(0.57) | <0.001* | 2.97(0.77,10.54) | 14(3.87) | 102(1.50) | <0.001* | 2.27(1.16,4.10) | 9(4.84) | 22(2.26) | 0.045 | 1.68(0.65,4.02) |
| Placenta previa, n(%) | 2(1.64) | 48(3.42) | 0.290 | 0.52(0.08,1.75) | 15(4.14) | 200(2.95) | 0.193 | 1.42(0.78,2.40) | 12(6.45) | 38(3.90) | 0.116 | 1.98(0.95,3.86) |
| Placenta previa with bleeding, n(%) | 2(1.64) | 12(0.86) | 0.383 | 1.74(0.27,6.57) | 10(2.76) | 66(0.97) | 0.001* | 3.80(1.81,7.17) | 9(4.84) | 9(0.92) | <0.001* | 8.47(3.15,23.13) |
| Premature delivery, n(%) | 36(29.51) | 63(4.49) | <0.001* | 8.45(5.11,13.87) | 94(25.97) | 323(4.76) | <0.001* | 6.71(5.06,8.85) | 58(31.18) | 58(5.95) | <0.001* | 6.66(4.34,10.25) |
| Cesarean section, n(%) | 77(63.12) | 661(47.08) | <0.001* | 1.77(1.19,2.66) | 208(57.46) | 3310(48.73) | 0.001* | 1.25(0.99,1.57) | 117(62.90) | 491(50.36) | 0.002* | 1.44(1.02,2.03) |
| Pelvic inflammation, n(%) | 13(10.66) | 91(6.48) | 0.079 | 1.89(0.96,3.48) | 59(16.30) | 399(5.88) | <0.001* | 2.45(1.75,3.38) | 40(21.51) | 100(10.26) | <0.001* | 1.97(1.26,3.01) |

**Abbreviation:** SAB, Spontaneous Abortion History; IA, Induced Abortion History; EP, Ectopic Pregnancy History; GDM: Gestational Diabetes Mellitus; PE: Preeclampsia; FGR: Fetal Growth Restriction; ICP: Intrahepatic Cholestasis of Pregnancy

^a.^Adjusting for factors including maternal age, PBMI, ART, nulliparity, age of first menstruation, menstrual cycle, menstrual duration, endocrine system diseases, history of smoking, and history of alcoholism.

*p < 0.05

**Table S8.** Clinical characteristics of women with SAB in twin versus singleton pregnancies.

| **Characteristic [N (%)]** | **Twin pregnancy**  **(n = 175)** | **Singleton pregnancy**  **(n = 1,202)** | **Statistic (χ2)** | **P.value** |
| --- | --- | --- | --- | --- |
| Age (Years) | 31.35[28.91,34.21] | 31.62[28.67,34.67] | 0.440 | 0.660 |
| PBMI, M (Q₁, Q₃) | 21.64[20.14,23.53] | 21.57[19.56,23.92] | -0.730 | 0.466 |
| ART, n(%) | 25(20.49) | 35(2.49) | 96.270 | <0.001* |
| Primigravida, n (%) | 100(81.97) | 864(61.54) | 20.135 | <0.001* |
| Age of first menstruation | 12.00[11.00,13.00] | 12.00[11.00,13.00] | -1.458 | 0.128 |
| Menstrual cycle | 28.00[28.00,29.00] | 28.00[28.00,30.00] | -0.698 | 0.447 |
| Menstrual duration | 5.00[4.00,6.00] | 5.00[4.00,6.00] | -2.041 | 0.035* |
| Endocrine system diseases | 31(25.41) | 407(28.99) | 0.702 | 0.402 |
| History of smoking, n(%) | 2(1.64) | 30(2.15) | 0.143 | 0.705 |
| History of alcoholism, n(%) | 27(22.50) | 229(16.44) | 2.887 | 0.089 |

**Abbreviation:** PBMI, Pre-pregnancy body mass Index; ; ART, Assisted reproductive technology. *p < 0.05

**Table S9.** Clinical characteristics of women with IA in twin versus singleton pregnancies.

| **Characteristic [N (%)]** | **Twin pregnancy**  **(n = 362)** | **Singleton pregnancy**  **(n = 6,792)** | **Statistic (χ2)** | **P.value** |
| --- | --- | --- | --- | --- |
| Age (Years) | 31.36[28.61,34.34] | 31.90[28.37,34.87] | 0.339 | 0.734 |
| PBMI, M (Q₁, Q₃) | 21.63[20.03,23.53] | 21.37[19.38,23.92] | -1.423 | 0.155 |
| ART, n(%) | 69(19.06) | 99(1.46) | 464.408 | <0.001* |
| Primigravida, n (%) | 260(71.82) | 3251(47.87) | 78.935 | <0.001* |
| Age of first menstruation | 12.00[11.00,13.00] | 12.00[11.00,13.00] | -2.331 | 0.014* |
| Menstrual cycle | 28.00[28.00,28.00] | 28.00[28.00,29.00] | 1.741 | 0.052 |
| Menstrual duration | 5.00[4.00,5.00] | 5.00[3.00,5.00] | -0.718 | 0.459 |
| Endocrine system diseases | 85(23.48) | 1769(26.05) | 1.177 | 0.278 |
| History of smoking, n(%) | 12(3.32) | 303(4.50) | 1.116 | 0.291 |
| History of alcoholism, n(%) | 56(15.73) | 1253(18.86) | 2.172 | 0.141 |

**Abbreviation:** PBMI, Pre-pregnancy body mass Index; ; ART, Assisted reproductive technology*p < 0.05

**Table S10.** Clinical characteristics of women with EP in twin versus singleton pregnancies.

| **Characteristic [N (%)]** | **Twin pregnancy**  **(n = 186)** | **Singleton pregnancy**  **(n = 975)** | **Statistic (χ2)** | **P.value** |
| --- | --- | --- | --- | --- |
| Age (Years) | 31.85[29.44,34.21] | 32.59[29.73,35.04] | 2.121 | 0.034* |
| PBMI, M (Q₁, Q₃) | 21.87[20.06,23.63] | 21.64[19.81,23.60] | -0.723 | 0.470 |
| ART, n(%) | 54(29.03) | 64(6.56) | 86.361 | <0.001* |
| Primigravida, n (%) | 152(81.72) | 645(66.15) | 17.586 | <0.001* |
| Age of first menstruation | 12.00[11.00,13.00] | 12.00[11.00,13.00] | -0.826 | 0.388 |
| Menstrual cycle | 28.00[28.00,28.00] | 28.00[28.00,28.00] | 0.333 | 0.712 |
| Menstrual duration | 7.00[5.00,7.00] | 6.00[5.00,7.00] | -1.467 | 0.125 |
| Endocrine system diseases | 38(20.43) | 219(22.46) | 0.374 | 0.541 |
| History of smoking, n(%) | 4(2.19) | 30(3.10) | 0.445 | 0.505 |
| History of alcoholism, n(%) | 44(24.18) | 158(16.46) | 6.259 | 0.012* |

**Abbreviation:** PBMI, Pre-pregnancy body mass Index; ; ART, Assisted reproductive technology. *p < 0.05

**Table S11.** The association between number of adverse obstetric history types and twin pregnancy.

| The number of adverse obstetric history types | **Univariate**  **analysis [OR(95%CI)]** | **P value** | **Multivariate analysis [aOR^a^(95%CI)]** | **P value** |
| --- | --- | --- | --- | --- |
| **0** |  |  |  |  |
| **1** | 0.557(0.493,0.629) | <0.001* | 0.798(0.642,0.997) | 0.045* |
| **2** | 0.925(0.742,1.142) | 0.480 | 1.087(0.807,1.459) | 0.581 |
| **3** | 2.432(1.376,4.069) | 0.001* | 2.453(1.248,4.581) | 0.007* |

**Abbreviation:** SAB, Spontaneous Abortion History; IA, Induced Abortion History; EP, Ectopic Pregnancy History。

^a.^Adjusting for factors including maternal age, PBMI, ART, nulliparity, age of first menstruation, menstrual cycle, menstrual duration, endocrine system diseases, history of smoking, and history of alcoholism.

**Table S12.** The impact of number of adverse obstetric history types on the risk of adverse perinatal outcomes in twin versus singleton pregnancies.

|  | **Twin pregnancy** | | | | | | | **Singleton pregnancy** | | | | | | |
| --- | --- | --- | --- | --- | --- | --- | --- | --- | --- | --- | --- | --- | --- | --- |
|  | **0[aOR^a^(95%CI)]** | **1[aOR(95%CI)]** | **p** | **2[aOR(95%CI)]** | **p** | **3[aOR(95%CI)]** | **p** | **0[aOR (95%CI)]** | **1[aOR (95%CI)]** | **p** | **2[aOR(95%CI)]** | **p** | **3[aOR(95%CI)]** | **p** |
| GDM | Reference | 1.27(0.79,2.07) | 0.335 | 1.43(0.78,2.66) | 0.250 | 11.45(3.19,54.72) | 0.001* | Reference | 0.87(0.77,0.97) | 0.014* | 0.98(0.82,1.17) | 0.806 | 2.62(1.50,4.63) | 0.001* |
| PE | Reference | 1.34(0.76,2.46) | 0.330 | 0.52(0.22,1.21) | 0.136 | 1.03(0.22,3.67) | 0.962 | Reference | 0.91(0.70,1.19) | 0.498 | 1.03(0.71,1.48) | 0.891 | 0.96(0.23,2.76) | 0.952 |
| FGR | Reference | 0.64(0.30,1.46) | 0.272 | 0.35(0.08,1.18) | 0.118 | 1.49(0.21,6.57) | 0.636 | Reference | 1.07(0.67,1.80) | 0.780 | 1.42(0.73,2.72) | 0.294 | 3.75(1.06,11.60) | 0.040* |
| ICP | Reference | 1.00(0.58,1.81) | 0.989 | 1.20(0.58,2.46) | 0.619 | 1.68(0.43,5.51) | 0.418 | Reference | 1.35(1.02,1.82) | 0.043* | 1.02(0.64,1.59) | 0.929 | 2.36(0.70,6.03) | 0.109 |
| Placenta accreta | Reference | 0.60(0.34,1.09) | 0.085 | 0.65(0.31,1.34) | 0.245 | 1.96(0.58,6.21) | 0.260 | Reference | 1.51(1.26,1.82) | <0.001* | 1.66(1.28,2.14) | <0.001* | 1.39(0.56,2.94) | 0.431 |
| Placenta accreta with bleeding | Reference | 0.67(0.22,2.47) | 0.498 | 0.82(0.18,3.62) | 0.781 | 4.11(0.68,22.43) | 0.102 | Reference | 1.51(0.96,2.46) | 0.088 | 0.84(0.38,1.76) | 0.658 | NA | 0.967 |
| Placenta previa | Reference | 3.02(0.57,55.86) | 0.296 | 7.07(1.15,136.22) | 0.075 | 15.80(1.39,358.89) | 0.030 | Reference | 1.11(0.82,1.52) | 0.504 | 1.58(1.05,2.38) | 0.028* | 1.56(0.37,4.42) | 0.470 |
| Placenta previa with bleeding | Reference | 2.11(0.36,40.07) | 0.491 | 5.11(0.71,102.87) | 0.154 | 15.99(1.35,372.75) | 0.032* | Reference | 1.06(0.63,1.87) | 0.838 | 1.18(0.52,2.54) | 0.686 | 6.05(1.37,18.89) | 0.005* |
| Premature delivery | Reference | 0.89(0.56,1.45) | 0.642 | 0.84(0.45,1.57) | 0.591 | 2.53(0.86,7.39) | 0.087 | Reference | 1.00(0.79,1.26) | 0.969 | 1.11(0.78,1.55) | 0.557 | 2.44(0.99,5.17) | 0.032* |
| Cesarean section | Reference | 0.48(0.29,0.76) | 0.002* | 0.30(0.16,0.55) | <0.001* | 0.91(0.28,3.59) | 0.887 | Reference | 1.02(0.93,1.13) | 0.652 | 0.97(0.83,1.13) | 0.575 | 0.77(0.46,1.29) | 0.321 |
| Pelvic inflammation | Reference | 1.27(0.70,2.42) | 0.455 | 0.73(0.31,1.68) | 0.466 | 0.87(0.13,3.63) | 0.863 | Reference | 0.90(0.74,1.09) | 0.268 | 1.01(0.75,1.35) | 0.936 | 1.59(0.68,3.25) | 0.241 |

**Abbreviation:** SAB, Spontaneous Abortion History; IA, Induced Abortion History; EP, Ectopic Pregnancy History; GDM: Gestational Diabetes Mellitus; PE: Preeclampsia; FGR: Fetal Growth Restriction; ICP: Intrahepatic Cholestasis of Pregnancy.

^a.^Adjusting for factors including maternal age, PBMI, ART, nulliparity, age of first menstruation, menstrual cycle, menstrual duration, endocrine system diseases, history of smoking, and history of alcoholism.

*p < 0.05

**Table S13.** Comparison of adverse perinatal outcomes associated with twin and singleton pregnancies in patients with different number of adverse obstetric history types.

|  | **1** |  |  |  | **2** |  |  |  | **3** |  |  |  |
| --- | --- | --- | --- | --- | --- | --- | --- | --- | --- | --- | --- | --- |
|  | **Twin pregnancy** | **Singleton pregnancy** | **p.value** | **aOR^a^(95%CI)** | **Twin pregnancy** | **Singleton pregnancy** | **p.value** | **aOR(95%CI)** | **Twin pregnancy** | **Singleton pregnancy** | **p.value** | **aOR(95%CI)** |
| GDM, n(%) | 164(39.52) | 2449(35.36) | 0.086 | 1.14(0.92,1.42) | 44(43.14) | 44(43.14) | 0.395 | 1.48(0.91,2.38) | 3(17.65) | 27(41.54) | 0.069 | 3.32(0.97,15.40) |
| PE, n(%) | 85(20.48) | 266(3.84) | <0.001* | 5.26(3.91,7.02) | 10(9.80) | 53(5.17) | 0.052 | 2.17(1.00,4.31) | 3(17.65) | 3(4.62) | 0.066 | 4.43(0.75,26.24) |
| FGR, n(%) | 26(6.27) | 88(1.27) | <0.001* | 4.22(2.53,6.78) | 3(2.94) | 18(1.76) | 0.399 | 1.65(0.38,4.99) | 2(11.77) | 3(4.62) | 0.273 | 2.76(0.34,18.10) |
| ICP, n(%) | 66(15.90) | 264(3.81) | <0.001* | 5.03(3.66,6.83) | 19(18.63) | 29(2.83) | <0.001* | 7.86(4.15,14.63) | 4(23.53) | 4(6.15) | 0.032* | 4.69(1.00,22.32) |
| Placenta accreta, n(%) | 62(14.94) | 690(9.96) | 0.001* | 1.31(0.96,1.77) | 18(17.65) | 119(11.61) | 0.075 | 1.56(0.88,2.66) | 6(35.29) | 7(10.77) | 0.014* | 3.32(0.97,15.40) |
| Placenta accreta with bleeding, n(%) | 12(2.89) | 112(1.62) | 0.050 | 1.27(0.62,2.38) | 4(3.92) | 10(0.98) | 0.010* | 3.76(1.00,11.73) | 3(17.65) | 0(0.00) | NA | NA |
| Placenta previa, n(%) | 11(2.65) | 191(2.76) | 0.897 | 1.02(0.50,1.84) | 6(5.88) | 43(4.20) | 0.426 | 1.43(0.53,3.22) | 2(11.77) | 3(4.62) | 0.273 | 2.76(0.34,18.10) |
| Placenta previa with bleeding, n(%) | 7(1.69) | 58(0.84) | 0.073 | 2.70(1.10,5.66) | 4(3.92) | 10(0.98) | 0.010* | 3.96(1.06,12.19) | 2(11.77) | 3(4.62) | 0.273 | 2.76(0.34,18.10) |
| Premature delivery, n(%) | 110(26.51) | 315(4.55) | <0.001* | 6.76(5.16,8.80) | 27(26.47) | 54(5.27) | <0.001* | 5.82(3.37,9.87) | 8(47.06) | 7(10.77) | <0.001* | 7.37(2.17,26.36) |
| Cesarean section, n(%) | 253(60.96) | 3360(48.51) | <0.001* | 1.62(1.31,2.02) | 55(53.92) | 506(49.37) | 0.380 | 1.13(0.75,1.73) | 13(76.47) | 30(46.15) | 0.026* | 3.79(1.20,14.60) |
| Pelvic inflammation, n(%) | 78(18.80) | 414(5.98) | <0.001* | 2.89(2.14,3.87) | 14(13.73) | 76(7.42) | 0.025* | 1.77(0.88,3.31) | 2(11.77) | 8(12.31) | 0.951 | 0.95(0.13,4.30) |

**Abbreviation:** SAB, Spontaneous Abortion History; IA, Induced Abortion History; EP, Ectopic Pregnancy History; GDM: Gestational Diabetes Mellitus; PE: Preeclampsia; FGR: Fetal Growth Restriction; ICP: Intrahepatic Cholestasis of Pregnancy

^a.^Adjusting for factors including maternal age, PBMI, ART, nulliparity, age of first menstruation, menstrual cycle, menstrual duration, endocrine system diseases, history of smoking, and history of alcoholism.

*p < 0.05

**Table S14.** Mediation analysis of twin pregnancy in the association between SAB and GDM outcomes.

|  | **Coefficient** | **S.E.** | ***p-*value** | **CI [2.5%]** | **CI [97.5%]** |
| --- | --- | --- | --- | --- | --- |
| Exposure-mediator effect | -0.016 | 0.099 | 0.870 | -0.211 | 0.178 |
| Mediator-outcome effect | -0.004 | 0.013 | 0.788 | -0.029 | 0.022 |
| Total effect | 0.035 | 0.013 | 0.006 | 0.010 | 0.059 |
| Direct effect | 0.035 | 0.013 | 0.006 | 0.010 | 0.059 |
| Indirect effect | 0.000 | 0.001 | 0.928 | -0.002 | 0.004 |

**Abbreviation:** S.E., Standard Error. *p < 0.05

**Table S15.** Mediation analysis of twin pregnancy in the association between SAB and PE outcomes.

|  | **Coefficient** | **S.E.** | ***p-*value** | **CI [2.5%]** | **CI [97.5%]** |
| --- | --- | --- | --- | --- | --- |
| Exposure-mediator effect | -0.016 | 0.099 | 0.870 | -0.211 | 0.178 |
| Mediator-outcome effect | 0.142 | 0.006 | 0.000 | 0.130 | 0.154 |
| Total effect | 0.003 | 0.006 | 0.591 | -0.008 | 0.015 |
| Direct effect | 0.003 | 0.006 | 0.566 | -0.008 | 0.015 |
| Indirect effect | -0.016 | 0.099 | 0.870 | -0.211 | 0.178 |

**Abbreviation:** S.E., Standard Error. *p < 0.05

**Table S16.** Mediation analysis of twin pregnancy in the association between SAB and ICP outcomes.

|  | **Coefficient** | **S.E.** | ***p-*value** | **CI [2.5%]** | **CI [97.5%]** |
| --- | --- | --- | --- | --- | --- |
| Exposure-mediator effect | -0.016 | 0.099 | 0.870 | -0.211 | 0.178 |
| Mediator-outcome effect | 0.132 | 0.006 | 0.000 | 0.120 | 0.143 |
| Total effect | -0.007 | 0.006 | 0.213 | -0.019 | 0.004 |
| Direct effect | -0.007 | 0.006 | 0.216 | -0.018 | 0.004 |
| Indirect effect | -0.002 | 0.013 | 0.824 | -0.027 | 0.022 |

**Abbreviation:** S.E., Standard Error. *p < 0.05

**Table S17.** Mediation analysis of twin pregnancy in the association between SAB and placental implantation outcomes.

|  | **Coefficient** | **S.E.** | ***p-*value** | **CI [2.5%]** | **CI [97.5%]** |
| --- | --- | --- | --- | --- | --- |
| Exposure-mediator effect | -0.016 | 0.099 | 0.870 | -0.211 | 0.178 |
| Mediator-outcome effect | 0.051 | 0.008 | 0.000 | 0.035 | 0.066 |
| Total effect | 0.013 | 0.007 | 0.085 | -0.002 | 0.027 |
| Direct effect | 0.013 | 0.007 | 0.083 | -0.002 | 0.027 |
| Indirect effect | -0.001 | 0.005 | 0.808 | -0.010 | 0.010 |

**Abbreviation:** S.E., Standard Error. *p < 0.05

**Table S18.** Mediation analysis of twin pregnancy in the association between SAB and premature delivery outcomes.

|  | **Coefficient** | **S.E.** | ***p-*value** | **CI [2.5%]** | **CI [97.5%]** |
| --- | --- | --- | --- | --- | --- |
| Exposure-mediator effect | -0.016 | 0.099 | 0.870 | -0.211 | 0.178 |
| Mediator-outcome effect | 0.205 | 0.007 | 0.000 | 0.192 | 0.218 |
| Total effect | 0.001 | 0.006 | 0.824 | -0.011 | 0.014 |
| Direct effect | 0.002 | 0.006 | 0.789 | -0.011 | 0.014 |
| Indirect effect | -0.003 | 0.021 | 0.852 | -0.043 | 0.037 |

**Abbreviation:** S.E., Standard Error. *p < 0.05

**Table S19.** Mediation analysis of twin pregnancy in the association between SAB and cesarean section outcomes.

|  | **Coefficient** | **S.E.** | ***p-*value** | **CI [2.5%]** | **CI [97.5%]** |
| --- | --- | --- | --- | --- | --- |
| Exposure-mediator effect | -0.016 | 0.099 | 0.870 | -0.211 | 0.178 |
| Mediator-outcome effect | 0.157 | 0.014 | 0.000 | 0.130 | 0.184 |
| Total effect | -0.007 | 0.013 | 0.607 | -0.033 | 0.019 |
| Direct effect | -0.007 | 0.013 | 0.616 | -0.032 | 0.019 |
| Indirect effect | -0.003 | 0.015 | 0.908 | -0.038 | 0.024 |

**Abbreviation:** S.E., Standard Error. *p < 0.05

**Table S20.** Mediation analysis of twin pregnancy in the association between IA and GDM outcomes.

|  | **Coefficient** | **S.E.** | ***p-*value** | **CI [2.5%]** | **CI [97.5%]** |
| --- | --- | --- | --- | --- | --- |
| Exposure-mediator effect | -0.792 | 0.064 | 0.000 | -0.918 | -0.666 |
| Mediator-outcome effect | -0.004 | 0.013 | 0.788 | -0.029 | 0.022 |
| Total effect | -0.041 | 0.007 | 0.000 | -0.055 | -0.026 |
| Direct effect | -0.041 | 0.007 | 0.000 | -0.056 | -0.027 |
| Indirect effect | 0.008 | 0.011 | 0.436 | -0.014 | 0.031 |

**Abbreviation:** S.E., Standard Error. *p < 0.05

**Table S21.** Mediation analysis of twin pregnancy in the association between IA and PE outcomes.

|  | **Coefficient** | **S.E.** | ***p-*value** | **CI [2.5%]** | **CI [97.5%]** |
| --- | --- | --- | --- | --- | --- |
| Exposure-mediator effect | -0.792 | 0.064 | 0.000 | -0.918 | -0.666 |
| Mediator-outcome effect | 0.142 | 0.006 | 0.000 | 0.130 | 0.154 |
| Total effect | -0.016 | 0.003 | 0.000 | -0.023 | -0.009 |
| Direct effect | -0.009 | 0.003 | 0.011 | -0.015 | -0.002 |
| Indirect effect | -0.112 | 0.013 | 0.000 | -0.135 | -0.085 |

**Abbreviation:** S.E., Standard Error. *p < 0.05

**Table S22.** Mediation analysis of twin pregnancy in the association between IA and ICP outcomes.

|  | **Coefficient** | **S.E.** | ***p-*value** | **CI [2.5%]** | **CI [97.5%]** |
| --- | --- | --- | --- | --- | --- |
| Exposure-mediator effect | -0.792 | 0.064 | 0.000 | -0.918 | -0.666 |
| Mediator-outcome effect | 0.132 | 0.006 | 0.000 | 0.120 | 0.143 |
| Total effect | -0.008 | 0.003 | 0.015 | -0.015 | -0.002 |
| Direct effect | -0.001 | 0.003 | 0.704 | -0.008 | 0.005 |
| Indirect effect | -0.104 | 0.012 | 0.000 | -0.129 | -0.082 |

**Abbreviation:** S.E., Standard Error. *p < 0.05

**Table S23.** Mediation analysis of twin pregnancy in the association between IA and placental implantation outcomes.

|  | **Coefficient** | **S.E.** | ***p-*value** | **CI [2.5%]** | **CI [97.5%]** |
| --- | --- | --- | --- | --- | --- |
| Exposure-mediator effect | -0.792 | 0.064 | 0.000 | -0.918 | -0.666 |
| Mediator-outcome effect | 0.051 | 0.008 | 0.000 | 0.035 | 0.066 |
| Total effect | 0.029 | 0.004 | 0.000 | 0.020 | 0.037 |
| Direct effect | 0.032 | 0.004 | 0.000 | 0.023 | 0.040 |
| Indirect effect | -0.044 | 0.008 | 0.000 | -0.062 | -0.030 |

**Abbreviation:** S.E., Standard Error. *p < 0.05

**Table S24.** Mediation analysis of twin pregnancy in the association between IA and premature delivery outcomes.

|  | **Coefficient** | **S.E.** | ***p-*value** | **CI [2.5%]** | **CI [97.5%]** |
| --- | --- | --- | --- | --- | --- |
| Exposure-mediator effect | -0.792 | 0.064 | 0.000 | -0.918 | -0.666 |
| Mediator-outcome effect | 0.205 | 0.007 | 0.000 | 0.192 | 0.218 |
| Total effect | -0.009 | 0.004 | 0.019 | -0.016 | -0.001 |
| Direct effect | 0.002 | 0.004 | 0.563 | -0.005 | 0.009 |
| Indirect effect | -0.163 | 0.017 | 0.000 | -0.194 | -0.130 |

**Abbreviation:** S.E., Standard Error. *p < 0.05

**Table S25.** Mediation analysis of twin pregnancy in the association between IA and cesarean section outcomes.

|  | **Coefficient** | **S.E.** | ***p-*value** | **CI [2.5%]** | **CI [97.5%]** |
| --- | --- | --- | --- | --- | --- |
| Exposure-mediator effect | -0.792 | 0.064 | 0.000 | -0.918 | -0.666 |
| Mediator-outcome effect | 0.157 | 0.014 | 0.000 | 0.130 | 0.184 |
| Total effect | 0.008 | 0.008 | 0.322 | -0.007 | 0.023 |
| Direct effect | 0.016 | 0.008 | 0.036 | 0.001 | 0.031 |
| Indirect effect | -0.127 | 0.015 | 0.000 | -0.163 | -0.102 |

**Abbreviation:** S.E., Standard Error. *p < 0.05

**Table S26.** Mediation analysis of twin pregnancy in the association between EP and GDM outcomes.

|  | **Coefficient** | **S.E.** | ***p-*value** | **CI [2.5%]** | **CI [97.5%]** |
| --- | --- | --- | --- | --- | --- |
| Exposure-mediator effect | 0.858 | 0.086 | 0.000 | 0.689 | 1.027 |
| Mediator-outcome effect | -0.004 | 0.013 | 0.788 | -0.029 | 0.022 |
| Total effect | -0.003 | 0.014 | 0.848 | -0.031 | 0.025 |
| Direct effect | -0.002 | 0.014 | 0.864 | -0.030 | 0.026 |
| Indirect effect | -0.003 | 0.012 | 0.908 | -0.030 | 0.017 |

**Abbreviation:** S.E., Standard Error. *p < 0.05

**Table S27.** Mediation analysis of twin pregnancy in the association between EP and PE outcomes.

|  | **Coefficient** | **S.E.** | ***p-*value** | **CI [2.5%]** | **CI [97.5%]** |
| --- | --- | --- | --- | --- | --- |
| Exposure-mediator effect | 0.858 | 0.086 | 0.000 | 0.689 | 1.027 |
| Mediator-outcome effect | 0.142 | 0.006 | 0.000 | 0.130 | 0.154 |
| Total effect | 0.019 | 0.007 | 0.006 | 0.005 | 0.032 |
| Direct effect | 0.007 | 0.007 | 0.327 | -0.007 | 0.020 |
| Indirect effect | 0.858 | 0.086 | 0.000 | 0.689 | 1.027 |

**Abbreviation:** S.E., Standard Error. *p < 0.05

**Table S28.** Mediation analysis of twin pregnancy in the association between EP and ICP outcomes.

|  | **Coefficient** | **S.E.** | ***p-*value** | **CI [2.5%]** | **CI [97.5%]** |
| --- | --- | --- | --- | --- | --- |
| Exposure-mediator effect | 0.858 | 0.086 | 0.000 | 0.689 | 1.027 |
| Mediator-outcome effect | 0.132 | 0.006 | 0.000 | 0.120 | 0.143 |
| Total effect | 0.018 | 0.007 | 0.006 | 0.005 | 0.031 |
| Direct effect | 0.007 | 0.007 | 0.286 | -0.006 | 0.020 |
| Indirect effect | 0.113 | 0.014 | 0.000 | 0.086 | 0.144 |

**Abbreviation:** S.E., Standard Error. *p < 0.05

**Table S29.** Mediation analysis of twin pregnancy in the association between EP and placental implantation outcomes.

|  | **Coefficient** | **S.E.** | ***p-*value** | **CI [2.5%]** | **CI [97.5%]** |
| --- | --- | --- | --- | --- | --- |
| Exposure-mediator effect | 0.858 | 0.086 | 0.000 | 0.689 | 1.027 |
| Mediator-outcome effect | 0.051 | 0.008 | 0.000 | 0.035 | 0.066 |
| Total effect | 0.060 | 0.008 | 0.000 | 0.044 | 0.076 |
| Direct effect | 0.056 | 0.008 | 0.000 | 0.040 | 0.072 |
| Indirect effect | 0.040 | 0.009 | 0.000 | 0.024 | 0.059 |

**Abbreviation:** S.E., Standard Error. *p < 0.05

**Table S30.** Mediation analysis of twin pregnancy in the association between EP and premature delivery outcomes.

|  | **Coefficient** | **S.E.** | ***p-*value** | **CI [2.5%]** | **CI [97.5%]** |
| --- | --- | --- | --- | --- | --- |
| Exposure-mediator effect | 0.858 | 0.086 | 0.000 | 0.689 | 1.027 |
| Mediator-outcome effect | 0.205 | 0.007 | 0.000 | 0.192 | 0.218 |
| Total effect | 0.039 | 0.007 | 0.000 | 0.024 | 0.053 |
| Direct effect | 0.021 | 0.007 | 0.003 | 0.007 | 0.035 |
| Indirect effect | 0.175 | 0.021 | 0.000 | 0.133 | 0.217 |

**Abbreviation:** S.E., Standard Error. *p < 0.05

**Table S31.** Mediation analysis of twin pregnancy in the association between EP and cesarean section outcomes.

|  | **Coefficient** | **S.E.** | ***p-*value** | **CI [2.5%]** | **CI [97.5%]** |
| --- | --- | --- | --- | --- | --- |
| Exposure-mediator effect | 0.858 | 0.086 | 0.000 | 0.689 | 1.027 |
| Mediator-outcome effect | 0.157 | 0.014 | 0.000 | 0.130 | 0.184 |
| Total effect | 0.026 | 0.015 | 0.082 | -0.003 | 0.055 |
| Direct effect | 0.013 | 0.015 | 0.398 | -0.017 | 0.042 |
| Indirect effect | 0.858 | 0.086 | 0.000 | 0.689 | 1.027 |

**Abbreviation:** S.E., Standard Error. *p < 0.05

**Figure legends**

**Figure S1** Causal diagram representing simple mediation. Legend: X: the exposure, M: the mediator, Y: the outcome, C: a set of confounders.

**
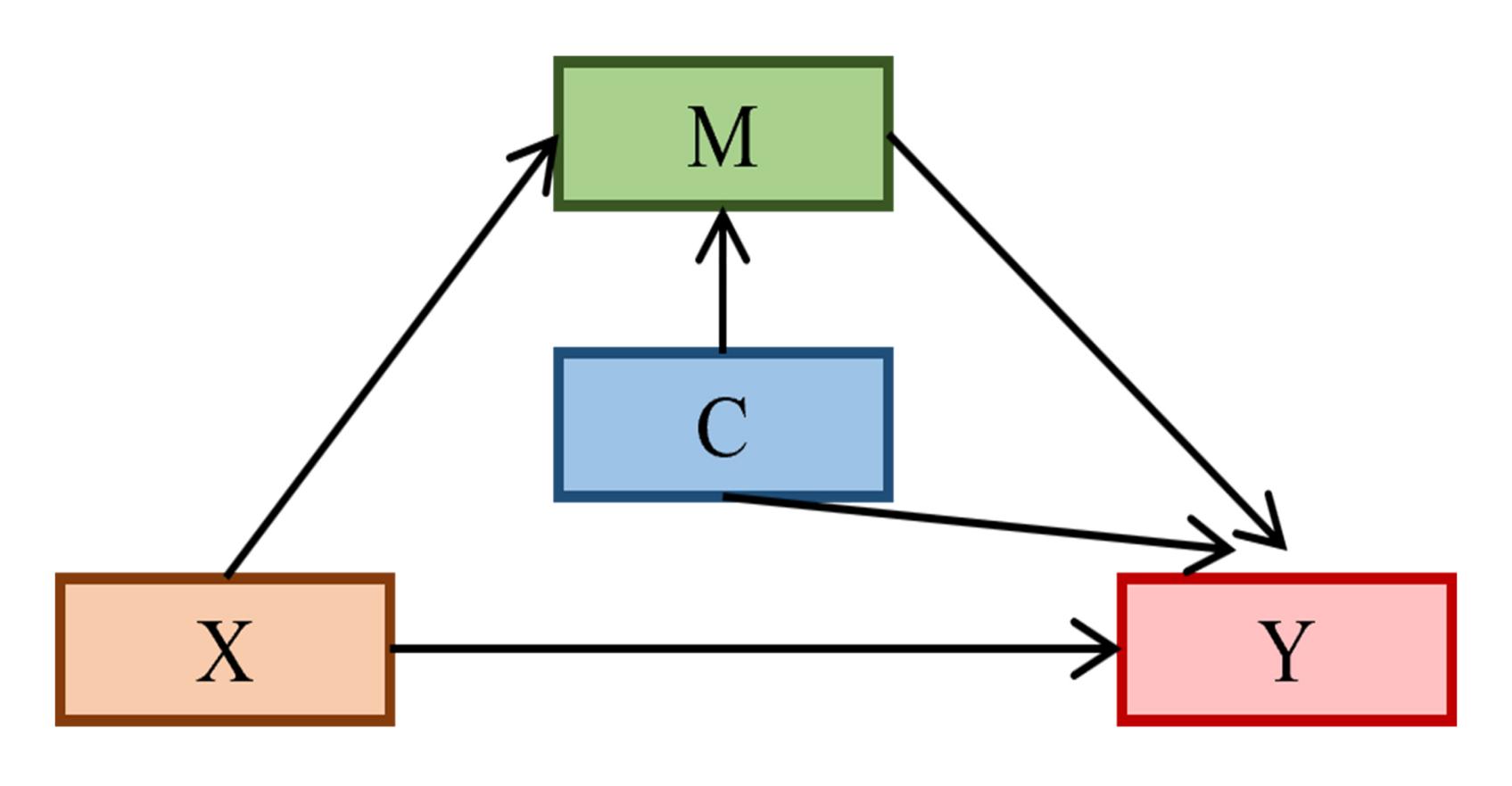
**
